# Environmental factors associated with nesting habits and age shape the composition and connection between skin and uropygial gland microbiomes of birds

**DOI:** 10.1111/1365-2656.70304

**Published:** 2026-06-30

**Authors:** Ester Martínez‐Renau, Kasun H. Bodawatta, Antonio M. Martín‐Platero, Manuel Martín‐Vivaldi, María Dolores Barón, Cristina Ruiz‐Castellano, Manuel Martínez‐Bueno, Knud A. Jønsson, Michael Poulsen, Juan José Soler

**Affiliations:** ^1^ Departamento de Ecología Funcional y Evolutiva Estación Experimental de Zonas Áridas (CSIC) Almería Spain; ^2^ Natural History Museum of Denmark University of Copenhagen Copenhagen Denmark; ^3^ Section for Molecular Ecology and Evolution Globe Institute, University of Copenhagen Copenhagen Denmark; ^4^ Departamento de Microbiología Universidad de Granada Granada Spain; ^5^ Unidad Asociada (CSIC): Coevolución: Cucos, Hospedadores y Bacterias Simbiontes Universidad de Granada Granada Spain; ^6^ Departamento de Zoología Universidad de Granada Granada Spain; ^7^ Department of Bioinformatics and Genetics Swedish Museum of Natural History Stockholm Sweden; ^8^ Section for Ecology and Evolution, Department of Biology University of Copenhagen Copenhagen Denmark; ^9^ Present address: Animal Ecology, Department of Ecology and Genetics Uppsala University Uppsala Sweden

**Keywords:** antimicrobial defences, avian microbiome, cavity nesting, pathogen exposure, symbiotic bacteria, uropygial gland secretion

## Abstract

1. Bacterial communities on skin and feathers can act as a critical line of defence against pathogenic infections in birds and may originate from secretions produced by the uropygial gland. These secretions reach the bird integument during preening, with the preening effort possibly determining the connectivity between uropygial gland and integument microbiomes.
2. The risk of pathogen infections depends on a number of variables, including environmental conditions (i.e. temperature and humidity), species identity, life‐history traits (i.e. cavity vs. open‐cup nesters) and life stage (i.e. age). Bacterial symbionts of the host, particularly those of the uropygial gland, may counter such pathogenic infections. We therefore hypothesise that bacterial communities of the uropygial gland differ among host species, age and nesting habits, with higher bacterial diversity in nestlings due to their immature immune system, and in cavity nesters due to potentially increased pathogen exposure.
3. We examined this using 16S rRNA metabarcoding of bacterial communities of the uropygial secretion (*N* = 352) and uropygial gland skin (*N* = 339) of nestlings and adults of 26 bird species from 14 families in southern Spain.
4. In accordance with the hypotheses, we find species‐specific differences in bacterial communities of uropygial gland skin and secretion, as well as an effect of age, with nestlings showing a higher bacterial diversity, especially in the uropygial gland skin. Additionally, the microbiotas of cavity‐nesting species are more diverse and heterogeneous than those of open nesters, with these effects more pronounced in adult and uropygial secretions. Finally, the uropygial gland is relatively larger in cavity‐ than in open‐nester species, which suggests that cavity nesters preen more often than the open nesters. Moreover, we found a stronger sharing of secretion and skin microbes in cavity nesters and nestlings compared to adults and open nesters.
5. Overall, our findings on the effects of age and nest type on structuring bird uropygial gland skin and secretion microbiota imply that age and pathogen risks related to nest environment could drive the external microbiome assembly in birds.

## Graphical abstract

This study provides new insights into how ecological conditions influence the evolution of host‐associated symbiotic communities and their functional roles. The findings suggest that pathogen pressures linked to nesting habits in birds shape bacterial communities of the uropygial gland, and their connection with the skin microbiome, where the antimicrobial defences act.

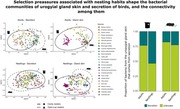

## 1 INTRODUCTION

Bacterial symbionts are ubiquitous across the animal tree of life (McFall‐Ngai et al., 2013), and both beneficial (mutualistic) and harmful (pathogenic and parasitic) symbionts significantly impact the ecology and evolution of hosts (Flint et al., 2012; Fraune & Bosch, 2010; McFall‐Ngai et al., 2013). Beneficial bacterial symbionts can provide protective functions against pathogens (Vorburger & Perlman, 2018), as bacterial communities on the skin, hair and feathers can act as a critical first line of defence against infections (Flechas et al., 2019; Javůrková et al., 2019; Longo & Zamudio, 2017). Such protection may involve beneficial bacteria residing within exocrine glands or growing in association with gland secretions that the hosts can then smear onto their bodies (Currie et al., 2006; Kaltenpoth et al., 2005; Martín‐Vivaldi et al., 2026; Soler et al., 2016). Therefore, these bacteria, or their antimicrobial compounds, must reach the integument (see examples in Flórez et al., 2015). This may be facilitated by host grooming (Martínez‐García et al., 2015), which may increase with increasing pathogen pressure (Fernández‐Marín et al., 2006).

In birds, the uropygial gland (a unique exocrine gland located on the upper rump) produces secretions with species‐specific chemical compositions (Jacob & Ziswiler, 1982; Mazorra‐Alonso, Peralta‐Sánchez, et al., 2024). The defensive properties of these secretions are likely partially mediated by gland‐associated bacterial symbionts (Bodawatta et al., 2020; Mazorra‐Alonso et al., 2021) that produce ectoparasite‐repellent volatiles (Mazorra‐Alonso, Peralta‐Sánchez, et al., 2024; Tomás et al., 2020) or antimicrobial substances (Seibel et al., 2024; Soler et al., 2008). During preening, birds spread these secretions along with beneficial bacteria to the skin, feathers and other body parts (Martín‐Vivaldi et al., 2014; Ruiz‐Rodríguez et al., 2009; Soler et al., 2016). As a result, the bacterial community of the uropygial gland is connected with bacterial communities in preened body locations (Martínez‐García et al., 2015; Soler et al., 2016).

Preening activities should be optimized according to pathogen exposures, which in turn may be driven by variation in nest types that affect the microclimate and microbial exposure of adults and their developing chicks. Cavity nesting, for instance, provides a partially isolated environment that is less exposed to environmental microbes than open‐cup nesting (Godard et al., 2007). Cavity nests also exhibit lower thermal variation, slightly reduced oxygen levels and higher carbon dioxide concentrations (Deeming & Mainwaring, 2015). Since these factors influence the composition of bacterial communities in avian nests, eggs and nestlings (West et al., 2015), it could be expected that cavity nesters show a less variable and compositionally distinct gland microbiota compared to open‐cup nesters.

Cavity nesting may, however, be associated with higher infection risks, because cavities frequently include potential pathogenic organisms from previous reproduction events (López‐Rull & Macías‐García, 2015; Peralta‐Sánchez et al., 2012). Cavity nesters indeed tend to exhibit stronger immune responses than open‐cup nesters (Møller et al., 2003, 2010; Møller & Erritzøe, 1996), and antimicrobial‐producing bacteria are more frequently found on the uropygial skin microbiota of cavity nesters than open‐cup nesters (Martínez‐Renau et al., 2022). Additionally, uropygial gland size is related to secretion volume (Martín‐Vivaldi et al., 2009; Møller et al., 2009; Ruiz‐Rodríguez et al., 2015), and both intra‐ and interspecific variations in gland size and secretion volume correlate with bacterial abundance and/or parasite prevalence (Galván & Sanz, 2006; Jacob et al., 2014; Magallanes et al., 2016; Møller et al., 2010; Moreno‐Rueda, 2010; Soler et al., 2012). Thus, uropygial gland size may serve as a proxy for pathogen exposure (see Pap et al., 2010); that is, if cavity nesters are exposed to higher pathogen pressures, they should have larger glands and preen more frequently than open‐cup nesters (but see Vincze et al., 2013). Since preening connects uropygial gland and integument microbiomes, cavity nesters are expected to show stronger connectivity between these two bacterial communities. Currently, we lack a comprehensive understanding of how the nest environment influences the composition of uropygial secretions and integument microbiomes.

Additionally, in contrast to adults, nestlings are in permanent contact with the nest environment and have not fully developed preen gland or immune system (Palacios et al., 2009; Stambaugh et al., 2011). Thus, the expected effect of nest type, either mediated by the risk of pathogen infection or by its isolation from the external environment, should vary for adults and nestlings. Consequently, as for gut microbiomes (Maraci et al., 2022; Zhou et al., 2020), we expect higher diversity and compositionally distinct bacterial communities of the uropygial gland skin and secretion in nestlings than in adults, in part depending on the nest environment. This prediction, as far as we know, has never been tested and would further support the importance of the environment in determining the age‐dependent composition of uropygial secretions and integument microbiomes.

Here, we characterized the diversity and composition of the bacterial communities of the uropygial gland skin and secretion in nestlings and adults of 26 European bird species and explored inter‐ and intraspecific variation in relation to species identity, age and nest type (cavity vs. open‐cup nesters). First, since gland size is considered a proxy for pathogenic risk and preening rate, we explored gland size differences between cavity and open nesters, with the prediction that cavity nesters, if experiencing higher pathogen pressures, should have larger glands. Second, since different species exploit different environments, experience different risks of pathogenic infection (Javůrková et al., 2019; Labrador et al., 2020; Peralta‐Sánchez et al., 2018) and produce uropygial secretions that may vary in chemical composition (Jacob & Ziswiler, 1982), we predict that both bacterial communities would differ between host species. Additionally, given the immature state of nestlings and their nest confinement, we expect higher bacterial diversity and different bacterial community compositions in nestlings than adults. Third, we considered two alternative predictions regarding nest type. Given that cavity nests are partially isolated from the external environment, we expect microbiomes of cavity nesters to be less diverse and more homogenous than those of open‐cup nesters. Moreover, this expected effect should be more pronounced in nestlings and skin microbiotas as they are more exposed to the nest environment. However, on the other hand, re‐used cavity nests can also be subject to higher pathogen pressures than open nests, potentially enhancing the evolution of antimicrobial defences, including immune responses and microbiological and chemical antagonistic characteristics of the uropygial secretion (Martínez‐Renau et al., 2022; Møller & Erritzøe, 1996). Under this scenario, cavity nesters should harbour more diverse microbiota (see Peralta‐Sánchez et al. (2012)) than open nesters, with stronger effects in nestlings due to their immature immune system. Finally, for a subset of species with available gland and secretion microbiomes, we performed connectivity and bacterial community source‐tracking analyses, predicting that microbiome connectivity and the rate of bacterial taxa sharing should be higher in cavity nesting than in open‐nesting birds.

## 2 MATERIALS AND METHODS

### 2.1 Study area, fieldwork and species

**FIGURE 1:**
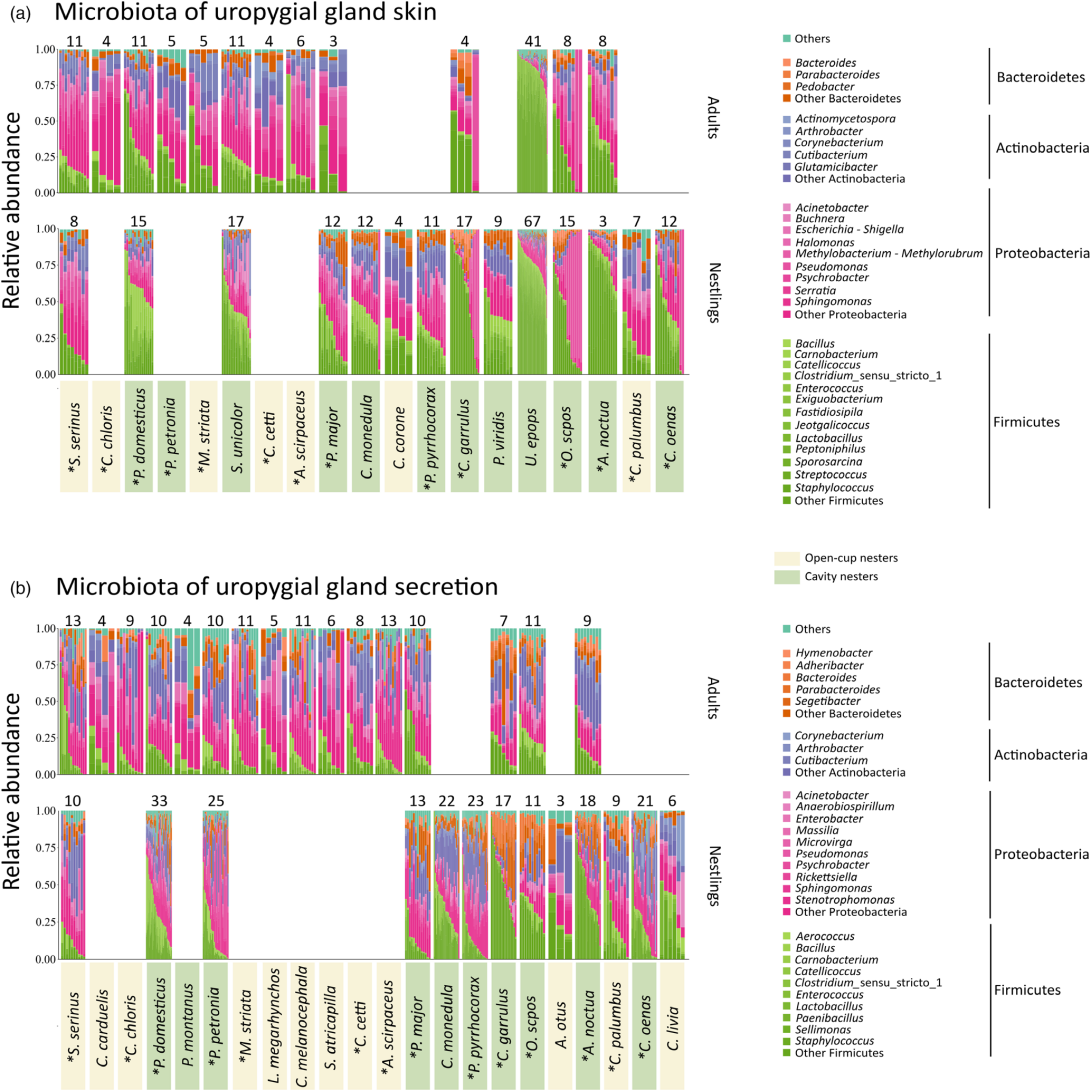
Stacked bar plot of the relative abundance of the most frequently detected genera in the uropygial gland skin (a) and secretion (b) for each individual, by bird species, age class and nesting type. Sample sizes are given above the bars. Bird species are ordered following phylogeny, and nesting type is shown with green (cavity nesters) and yellow labels (open‐cup nesters). Species considered in the source‐Stracking analyses are marked with an*.

Fieldwork was carried out during the breeding seasons (March to July) of 2018, 2019 and 2021 at two localities in southern Spain: the Hoya de Guadix (37°15′ N; 3°01′ W), a semiarid high‐altitude plateau, and the Charca de Suárez, a wetland by the coast in Motril (36°43′18.707″ N, 3°32′30.836″ W). We sampled individuals from 26 species belonging to 14 families and six orders (Figure 1).

From mid‐March until mid‐June, we visited the nest boxes in the area every 3 days and searched intensively for nests of open‐cup‐nesting species until egg detection, which allowed us to infer the laying date and, hence, to plan the sampling date of adults and nestlings. We sampled nestlings during the last quarter of the nestling period or a few days after they abandoned the nest. Most cavity‐nesting adults were captured from the nest box during incubation (nest box internal dimensions: 18 × 21 × 35 cm and 24 cm from the bottom to the hole entrance). To maximise the number of sampled species, we set up mist nets once in the Hoya de Guadix and twice in the Charca de Suárez. Mist nets were opened at sunrise and closed approximately 5 h later.

The uropygial gland skin was sampled by rubbing the surface skin, including the opening, with a sterile cotton swab (APTACA, ref. 2160, Canelli, Italy) wetted in sterile Phosphate Buffered Saline (1X PBS, 0.2 M). The swab was kept in a sterile microfuge vial with 1 mL of sterile PBS. The uropygial gland secretion was extracted after cleaning the skin of the gland with 70% ethanol, using a sterile micro‐capillary (32 mm, 10 μL; BlauBrand, ref. 7091 18) placed on the gland opening, while slightly pressing the gland. Once the gland was emptied, the micro‐capillary was placed in a sterile microfuge vial. Uropygial gland skin and secretion samples were stored at −18°C until DNA extraction. We also measured body mass with a digital scale (accuracy 0.01 g) and uropygial gland dimensions (length, width and height) with a digital calliper (accuracy 0.01 mm) following Martín‐Vivaldi et al. (2009). We calculated the gland volume as the width × height × length and log10 transformed before the statistical analyses to adjust the values to a normal distribution. Finally, we ringed all individuals with numbered metal rings (Ministerio de Agricultura, Spain) to avoid resampling. We also collected nine field controls by holding the swab wetted in PBS, or the micro‐capillary, in the air for a few seconds, after which they were placed in microfuge tubes. Field controls were processed the same as samples and used to detect possible contaminants. This study was reviewed and approved by the Environmental Department of the Regional Government of Andalucía, Spain (Consejería de Agricultura, ganaderia, Pesca y Desarrollo Sostenible, reference DGPAG/SA/SIS).

### 2.2 DNA extraction and amplicon sequencing

DNA from the uropygial gland skin bacterial communities was extracted using the FavorPrep™ Blood Genomic DNA Extraction Mini Kit (Favorgen Biotech Crop., Taipei, Taiwan), with the following protocol: first, we sonicated the sample for 2 min at 120 Hz to release the bacterial cells from the swab. We then removed the swab and centrifuged the PBS with the bacteria at 13,000 rpm for 5 min. Afterwards, we discarded the supernatant and added to the pellet 180 μL of TES (25 mM Tris–HCl, pH 8, 10 mM EDTA and 10% sucrose), 10 mg/mL of lysozyme, and 4 μL of RNase (10 mg/mL). Subsequent steps were performed following the FavorPrep™ instructions. DNA extractions from the bacterial communities within uropygial secretions were performed following the protocol in Boom et al. (2000) with the subsequent modifications to optimise lysis and DNA extraction. The sample was resuspended in 30 μL of lysis solution and incubated for 10 min at 75°C. Afterwards, a volume of 30 μL of neutralisation solution was added. We included one extraction blank per 100 samples.

Extracted DNA was sent to the facilities of the Institute of Parasitology and Biomedicine ‘López‐Neyra’ (IPBLN, Granada, Spain) where the library preparation and sequencing on an Illumina MiSeq platform were carried out. Briefly, the 16S rRNA libraries corresponding to the V6–V8 variable region were constructed by a two‐step amplification approach, each PCR step followed by a purification step. In the first step, the primer pair B969F (ACGCGHNRAACCTTACC) and BA1406R (ACGGGCRGTGWGTRCAA) (Comeau et al., 2011) was used, which targets the V6–V8 16S variable region with partial overlap of Illumina primers, as previously described (Preheim et al., 2013). In the second step, the first PCR was re‐amplified a few cycles to introduce a unique combination of two Illumina barcodes for each sample.

### 2.3 Bioinformatics analyses

Raw reads from uropygial gland skin and secretion were processed within QIIME2 v2020.6 (Bolyen et al., 2019) environment. We used the DADA2 (Callahan et al., 2016) plugin for primer trimming, sequence quality filtering, chimaera removal and Amplicon Sequence Variants (ASV) clustering. Then, the representative sequence of each ASV was taxonomically assigned using the Silva 138 database (Quast et al., 2012). Non‐bacterial sequences, including mitochondria, chloroplast and sequences not assigned to any domain, were removed. Potential contaminant ASVs were identified (and removed) by comparing samples and field controls with the Decontam package in R (Davis et al., 2018; R‐Core‐Team, 2020), using the prevalence method with a threshold of 0.4. A sequence alignment and a bacterial phylogeny (rooted at its midpoint) were generated by using the function ‘align‐to‐tree‐mafft‐fasttree’ in QIIME2.

### 2.4 Statistical analyses

Given the difficulties in finding nests and sampling wild birds, our dataset does not include samples from both adults and nestlings for all bird species, which limited the possibility of performing statistical analyses that incorporate every factor of interest in the same model (i.e. impossibility of estimating interactions accurately). Therefore, for each analysis, we selected the samples that allowed us to best test each of our predictions. All analyses were performed in R v4.0.2 (R Core Team, 2020). An overview of the hypothesis tested and the statistical models used is provided in Table 1.

**TABLE 1:** Overview of hypotheses, statistical models, sample types and sample sizes by model.

Variables tested	Question	Models used				Samples used	Sample size
Effect of nest type on gland size	Is the uropygial gland larger in cavity than open‐cup nesters?		Adults		Linear models	Adult samples	Cavity: 127 Open‐cup: 100
			Nestlings		Linear mixed models	Nestling samples	Cavity: 354 Open‐cup: 38
Effect of age and species on the microbiota	Do species identity and age class affect the diversity and composition of bacterial communities on the gland skin and gland secretions?	Alpha diversity	Secretion		Linear mixed models	Species with secretion samples from adults and nestlings	Adults: 70 Nestlings: 127
			Gland skin		Linear mixed models	Species with gland skin samples from adults and nestlings	Adults: 97 Nestlings: 164
		Beta diversity	Secretion		PERMANOVAs	Species with secretion samples from adults and nestlings	Adults: 70 Nestlings: 127
			Gland skin		PERMANOVAs	Species with gland skin samples from adults and nestlings	Adults: 97 Nestlings: 164
Effect of nest type (cavity vs. open‐cup nesters) on the microbiota	Do cavity nesters have less diverse and more homogeneous bacterial communities than open‐cup nesters?	Alpha diversity	Secretion	Adults	Linear models	Adult samples	Cavity: 61 Open‐cup: 80
				Nestlings	Linear mixed models	Nestling samples	Cavity: 183 Open‐cup: 28
			Gland skin	Adults	Linear models	Adult samples	Cavity: 91 Open‐cup: 30
				Nestlings	Linear mixed models	Nestling samples	Cavity: 200 Open‐cup: 18
		Beta diversity	Secretion	Adults	PERMANOVAs	Adult samples	Cavity: 61 Open‐cup: 80
				Nestlings	PERMANOVAs	Nestling samples	Cavity: 183 Open‐cup: 28
			Gland skin	Adults	PERMANOVAs	Adult samples	Cavity: 91 Open‐cup: 30
				Nestlings	PERMANOVAs	Nestling samples	Cavity: 200 Open‐cup: 18
Connectivity (correlation) between secretion and gland skin microbiotas	Is there a correlation (connectivity) between gland skin and secretion microbiotas?		Adults		Mantel tests	Adults with samples for both secretion and gland skin communities	Secretion: 44 Gland skin: 44
			Nestlings		Mantel tests	Nestlings with samples for both secretion and gland skin communities	Secretion: 35 Gland skin: 35
Effect of age and species on the source‐tracking results	Does the bacterial community of the gland skin derive from gland secretions, especially in cavity‐nesting species and nestlings?				Linear mixed models	Individuals with samples for both secretion and gland skin communities	Adults: 23 Nestlings: 21
Effect of nest type on the source‐tracking results			Adults		Linear models	Adults with samples for both secretion and gland skin communities	Cavity: 18 Open‐cup: 26
			Nestlings		Linear mixed models	Nestlings with samples for both secretion and gland skin communities	Cavity: 25 Open‐cup: 11

#### 2.4.1 Gland size and association with nest type

To test for effects of nest type on the gland size, we conducted standardized major axis (SMA) regressions to control for interspecific variability in gland volume by tarsus length and then used residuals, separately estimated for adults and nestlings, in the statistical analyses. The linear model (LM) for adults included nest type, the species ID nested within nest type and sampling locality as fixed factors. The linear mixed model (LMM) for nestlings also included information on nest identity as a random factor.

#### 2.4.2 Alpha and beta diversity indexes

Due to the complexity of our dataset (i.e. including different species, ages and body locations), read depths varied greatly among samples. To avoid excluding informative samples, even those with relatively few reads, we rarefied to the minimum sampling depth (1651). We did so using the ‘rarefy_even_depth’ function in the ‘phyloseq’ package (McMurdie & Holmes, 2013) in R v4.0.2 (R‐Core‐Team, 2020). We calculated the Chao1 richness and the Shannon diversity (evenness) using the ‘microbiome’ package (Lahti & Shetty, 2017), and the Faith's phylogenetic diversity (PD) with the ‘picante’ package (Kembel et al., 2010). We used the ‘phyloseq’ package (McMurdie & Holmes, 2013) to calculate beta diversity distance matrices. Since the alpha diversity indexes did not follow a normal distribution, we applied a Box‐Cox transformation by calculating the optimal lambda parameter for each variable. We used the transformed alpha diversity variables for subsequent analyses. We calculated Bray–Curtis and weighted UniFrac that account for relative abundances, and Jaccard and unweighted UniFrac that treat all ASVs equally regardless of abundance. In addition, UniFrac metrics incorporate the phylogenetic relationships among bacterial taxa. We also calculated beta diversity after applying to the unrarefied ASV table the Centered Log‐Ratio (CLR) transformation with the ‘microbiome’ v1.20.0 package (Lahti & Shetty, 2017) and the Phylogenetic Isometric Log‐Ratio (PhILR) transformation using the ‘philr’ v1.24.0 package (Silverman et al., 2017). However, since with a few exceptions, PERMANOVA results did not differ qualitatively (i.e. similar statistical inferences, see ESM Table S1), we reported our findings using the rarefied ASV table (see, Schloss, 2024).

#### 2.4.3 Testing the effect of age class, species identity and nest type on alpha and beta diversities

We used LMMs to test for the effects of age class, species ID and nest type, separately for gland skin and secretion samples, on bacterial alpha diversity indexes using *aov* and *lmer* functions from ‘stats’ (R‐Core‐Team, 2020) and ‘lme4’ (Bates et al., 2015) R packages respectively. We first tested the effect of age (adults vs. nestlings) and species identity (ID) using species with samples for both age classes. In order to control for the effect of sampling locality, we included it as a fixed factor (two levels, Guadix and Charca de Suárez) in the models. The models also included nest identity as the random factor (Species + Age + Locality + (1 | Nest)), while the interaction between the fixed factors was explored in separate models that also included the main effects.

The effects of nest type on alpha diversities were explored in separate models for adults and nestlings, which allowed us to increase the number of species included in the analyses. The models also included species ID (nested within nest type) and sampling locality as fixed factors, and, in the case of nestling samples, nest ID as a random factor (Nest type/Species + Locality + (1|Nest)).

Since nest type have a strong phylogenetic component, we controlled the alpha diversity analyses for the bird phylogeny (Figure S1; for an explanation of how the bird phylogeny was built, see Supporting Information) by means of Bayesian phylogenetic mixed models (MCMCglmm). The phylogeny was used to estimate the predicted effects of nest type on bacterial community diversity using the MCMCglmm package (Hadfield, 2010) in R v4.0.2 (R Core Team, 2020), after controlling for the effect of locality, which was also included in the models. The model was run using the Gaussian family, an uninformative prior, 2000,000 iterations, a 100,000 burn‐in period and a thinning interval of 2000. We also used Geweke's convergence diagnostic for Markov chains (Geweke, 1992) with all but one of the *z*‐scores between the critical values of −1.96 and 1.96. The phylogenetic effect was reported as heritability (*h* ^2^) (Hadfield, 2010), which is a measure of phylogenetic signal that ranges from zero (no signal) to one (high signal, Pearse et al., 2025).

To test for the effect of age class, species ID and nest type on beta diversity, we performed Permutational MANOVAs (PERMANOVAs) using *adonis2* from the package ‘vegan’ (Oksanen et al., 2022). We tested the effects of age and species ID by including both variables as fixed factors in the models, while restricting permutations to nest ID through the strata parameter. The effects of nest type were also explored, including nest type and species ID nested within nest type as independent variables (Nest type/Species), while the nestling models also included nest ID (Nest type/Species/Nest). Sampling locality was included in the models as for the alpha diversity analyses. Bacterial community differences were visualised with Non‐Metric Multidimensional Scaling (NMDS) plots (for Bray–Curtis distances) using the ‘phyloseq’ package. Furthermore, we calculated betadispersion for all the combinations of age and nest type, using the function *betadisper* from the R package ‘vegan’ (Oksanen et al., 2022). To explore the effects of the nest type on beta diversity while considering the bird phylogeny, we performed separate Mantel tests for adults and nestlings using distance matrices for the bacterial communities of the uropygial gland skin or secretion as dependent variables. Independent matrices included a binary matrix with information on the nest type (i.e. same (0) or different (1) nest type), a matrix with information on sampling locality and a matrix with phylogenetic distances between bird species. When analysing nestlings, we also included a binary matrix indicating whether nestlings belonged to the same (0) or different (1) nests.

Finally, to explore whether the expected effects of age on alpha diversity indexes differed for skin and secretion samples, or whether the effect size of nest type varied depending on age or sampled body site, we calculated and compared values of Cohen's *d* (i.e. effect size) associated with the factors and estimated in the statistical models. We adopted this approach because the structure of our dataset prevented us from exploring effects of all factors of interest in the same model without losing samples and, hence, statistical power (see above). Comparing effect sizes allows us to infer whether the effects of one factor (i.e. nest type) depended on others (i.e. location sampled or age). As far as we are aware, there are no standardized effect size metrics for PERMANOVA analyses and we therefore reported *R* ^2^ values from the adonis2 function as proxies for effect sizes. These values are descriptive and no strong conclusions can be drawn from them, as they are not standardized metrics that can be compared across datasets.

#### 2.4.4 Testing the effect of fledging

As some of the nestling samples were collected shortly after leaving the nests and the microbiomes of fledglings may differ from those of nestlings, we tested the main analyses excluding the fledgling samples (23 individuals from two species). Statistical inferences did not vary when fledglings were excluded (ESM Tables S2 and S3) and therefore, we conducted all our analyses, including both nestlings and fledglings.

#### 2.4.5 Connectivity between uropygial gland secretion and skin microbiomes

For these analyses, we used a subset of 80 individuals from 14 species belonging to 10 families and four orders, for which we had both uropygial gland skin and secretion microbiomes. Of these, 43 individuals were cavity nesters (8 species; 24 nestlings and 19 adults), while 37 were open‐cup nesters (6 species; 11 nestlings and 26 adults) (Figure 1).

To test for associations between bacterial communities of the two body locations, we used partial Mantel tests with 10,000 permutations. For these models, we used the distance matrix of the uropygial gland skin microbiome as the dependent variable, and that of the uropygial secretion as the independent variable. We added a binary matrix where each value indicated whether a cell corresponded to an interaction between individuals of the same (0) or different (1) species. Mantel tests were performed on the whole set of samples and for cavity and open‐cup nesters separately, allowing us to explore whether correlations between bacterial communities were consistent across levels of early‐life environmental conditions. We used several methods to estimate and build the distance matrix (Bray–Curtis, Jaccard, weighted UniFrac and unweighted UniFrac) and repeated the analyses after collapsing the ASV table to the genus level. We also controlled these analyses for the host phylogeny (Figure S1) by exchanging the species matrix with phylogenetic distances between bird species. We used the 95% confidence interval (CI) of the Mantel r to estimate effect sizes.

To explore whether the bacterial community in the uropygial gland skin originated from the bacterial community inhabiting the uropygial gland, we conducted source‐tracking analysis using the FEAST package (Shenhav et al., 2019) in R v4.0.2 (R‐Core‐Team, 2020). We categorised the secretion bacterial community as the ‘source’, and the skin bacterial community as the ‘sink’. For each individual, we estimated the proportion of bacteria in the uropygial gland skin that may have originated from the gland secretion. Subsequently, we explored whether the proportion of the uropygial gland bacterial community found on the skin differed among age classes, species and nest type using the models described above for the alpha diversity.

## 3 RESULTS

### 3.1 Microbiome communities of uropygial gland skin and secretion

We successfully sequenced bacterial communities of 339 uropygial gland skin samples (121 from adults and 218 from nestlings) and 352 uropygial gland secretion samples (148 from adults and 204 from nestlings, Figure 1). Both data sets included cavity (skin: 12 species; secretion: 10 species) and open‐cup (skin: 7 species; secretion: 12 species) nesters (Figure 1). All cavity‐nesting species were secondary cavity nesters and were sampled in artificial nest boxes previously used by the same or different species.

From the uropygial gland skin communities, we obtained 10,077,955 sequences (average ± SD: 29,728.5 ± 20,876.3) classified into 23,101 ASVs. Rarefying these samples led to 15,981 ASVs (ESM Table S4). The bacterial community of the gland skin of birds was dominated by Firmicutes (51.7%), Proteobacteria (29.9%), Actinobacteria (10.5%) and Bacteroidetes (5.3%) (Figure 1). We obtained 8,223,291 bacterial sequences (23,361.6 ± 16,269; 33,658 ASVs) from uropygial gland secretion samples, retaining 25,921 ASVs after rarefying (ESM Table S5). Secretion communities were dominated by Proteobacteria (33.9%), Firmicutes (24.2%), Actinobacteria (22.5%) and Bacteroidetes (13.2%) (Figure 1). Notably, *Staphylococcus* was more abundant in the gland skin of cavity (mean ± 95% CI = 6.58% ± 7.10) than open‐cup nesters (mean ± 95% CI = 3.77% ± 3.94), while *Stenotrophomonas* and *Enterobacter* were more abundant in secretions from open‐cup‐nesting species than any other group (mean ± 95% CI open‐cup nester secretions >3.46% ± 4.46; mean ± 95% CI other groups <1.78% ± 2.11) (Figure S2). The most abundant genus on the gland skin, *Pseudomonas*, was widespread across the host phylogeny, while other bacteria were particularly abundant in certain host taxa. This was, for instance, the case for *Fastidiosipila*, *Peptoniphilus*, and two undetermined genera of the family Lachnospiraceae that were mainly found in hoopoes (Figure S2).

### 3.2 Is the uropygial gland larger in cavity than open‐cup nesters?

Nest type was associated with uropygial gland size. After controlling for tarsus length, species identity and sampling locality, adults and nestlings of cavity‐nester species had larger uropygial glands than open‐cup nester species (adults: *F* = 18.15, *p* < 0.001; LS means ± SE cavity nesters = 0.003 ± 0.07; LS means ± SE open‐cup nesters = −0.13 ± 0.07; nestlings: *F* = 5.41, *p* = 0.021; LS means ± SE cavity nesters = 0.05 ± 0.09; LS means ± SE open‐cup nesters = −0.004 ± 0.08).

### 3.3 Do species identity and age class affect the diversity and composition of bacterial communities on the gland skin and gland secretions?

**FIGURE 2:**
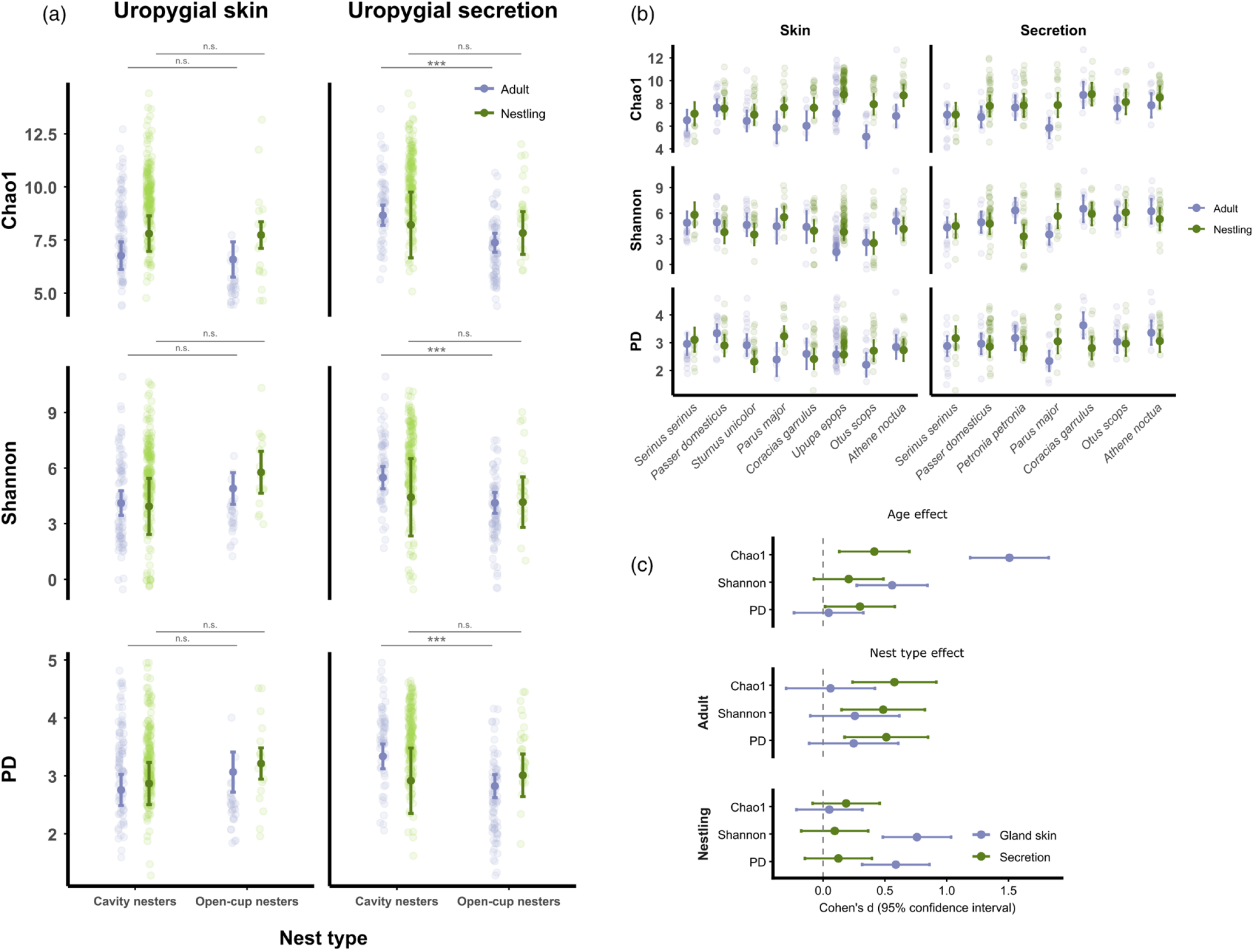
Bacterial community of the uropygial secretion of cavity nesters is richer and more diverse than that of open‐cup nesters. (a) Least square means (±95% confidence interval [CI]) of Box‐Cox transformed alpha diversity indexes (Chao1, Shannon index and PD) for cavity and open‐cup nesters, in adults and nestlings, after controlling for species identity and sampling locality. Statistically significant comparisons are labelled with ***(<0.001). Faded dots represent individual raw data points after transforming the variables with Box‐Cox transformation. (b) Least square means (±95% CI) of Box‐Cox transformed alpha diversity indexes (Chao1, Shannon index and PD) for adults and nestlings after controlling for species identity and locality. Faded dots represent individual raw data points after transforming the variables with Box‐Cox transformation. (c) Cohen's d estimates of effect size and 95% CIs for factors age and nest type.

The bacterial communities of the gland skin and secretion of nestlings were richer (Chao1 estimate; *F* > 7.38, *p* < 0.007) than those of adults (Figure 2b, ESM Table S6). Shannon diversity indices of skin (*F* = 16.67, *p* < 0.001) but not secretion (*F* = 1.81, *p* = 0.180) were significantly higher in nestlings than in adults (Figure 2b, ESM Table S6). PD values did not differ between adults and nestlings for either skin or secretion communities (*F* < 3.82, *p* > 0.052; Figure 2b, ESM Table S6). Moreover, the effect sizes (Cohen's *d*) of age on Chao1 and Shannon were significantly larger in the bacterial community of the uropygial skin than in the secretion (95% CI Chao1 gland skin 1.19–1.83 and gland secretion 0.13–0.70; 95% CI Shannon gland skin 0.27–0.84 and gland secretion (−0.07)–0.49; Figure 2c, ESM Table S6). Richness and diversity also varied across bird host species for the gland skin (*F* > 3.32, *p* < 0.003; ESM Table S6), and the interaction between age and species was significant for secretion and skin samples (*F* > 3.47, *p* < 0.002), with the exception of Chao1 index for the secretion, suggesting that the significant effect of age was host‐species‐dependent (Figure 2b). Similarly, the composition of the bacterial community of gland skin and secretion differed between species and ages (*F* > 1.89, *p* < 0.001; except for weighted UniFrac for gland skin), and there was a significant interaction between them (*F* > 1.54, *p* < 0.001; ESM Table S7). In this case, age apparently explained a similar portion of the variation in the composition of bacterial communities of the uropygial skin and gland secretion (ESM Table S7).

### 3.4 Do cavity nesters have less diverse and more homogeneous bacterial communities than open‐cup nesters?

The bacterial communities of the secretion, but not of the gland skin, of cavity‐nesting adults were richer and more diverse than those of open‐cup nesters for all the considered alpha diversity indexes (*F* > 13.85, *p* < 0.001; Figure 2a and ESM Table S6). In nestlings, the effect of nest type on the diversity of bacterial communities was not statistically significant when considering either those of the gland skin or of the secretion (*F* < 2.99, *p* > 0.086; Figure 2a and ESM Table S6). Moreover, for the secretion bacterial community, the effect of nest type was more clearly detected in adults than in nestlings (Cohen's d 95% CI Chao1 adults 0.24–0.92 and nestlings (−0.09)–0.46; 95% CI Shannon adults 0.15–0.82 and nestlings (−0.18)–0.37; 95% CI PD adults 0.17–0.85 and nestlings (−0.15)–0.39; Figure 2c and ESM Table S6). The statistical inferences for adults and nestlings did not change after controlling for the bird phylogeny (ESM Table S8).

**FIGURE 3:**
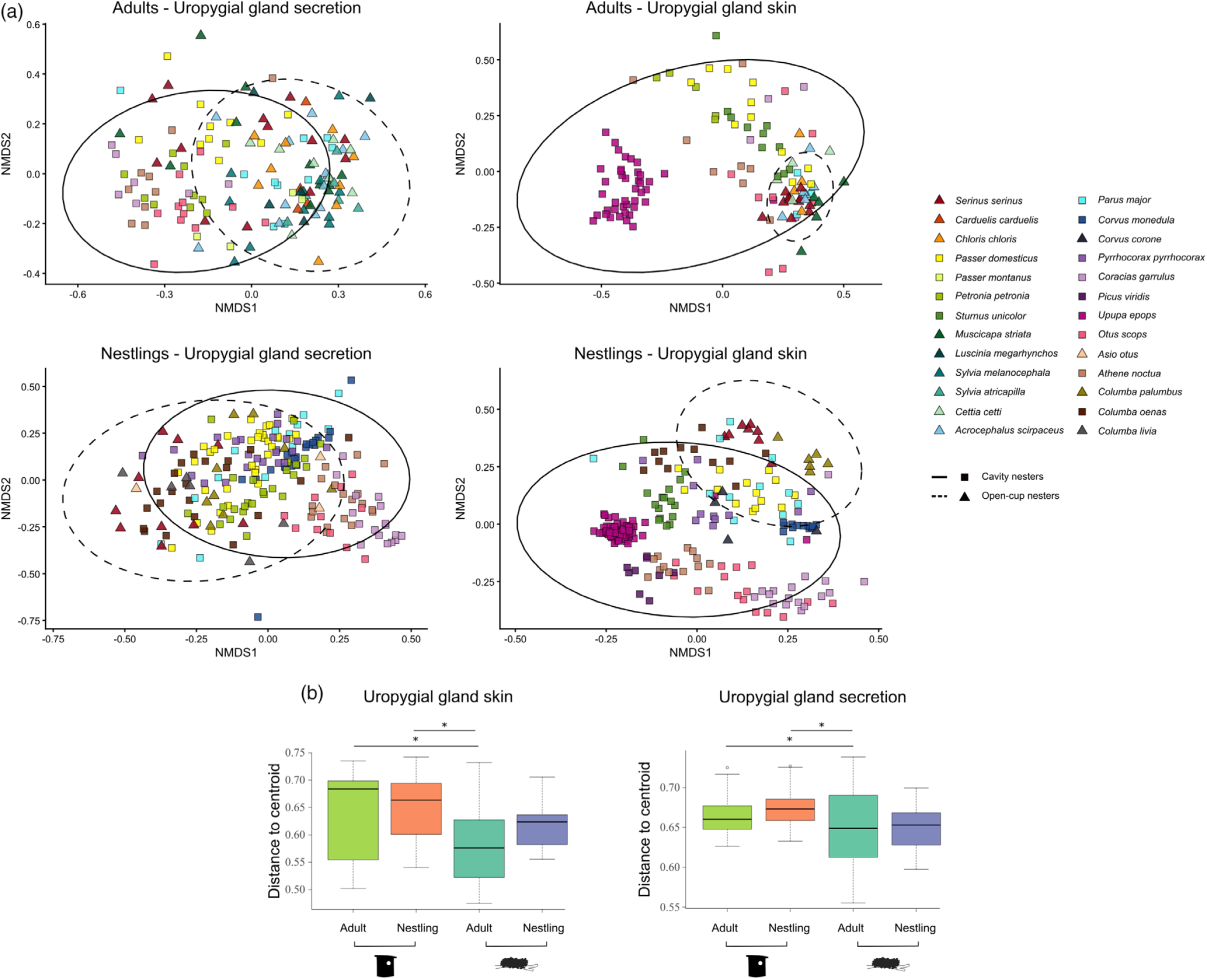
Nest type influences the bacterial composition of adults and nestlings. (a) NMDS ordination plots of adults and nestlings showing the diversity of bacterial communities of uropygial gland skin and secretion for the sampled species, including cavity (squares) and open‐cup nesters (triangles). Ellipses correspond to 95% confidence intervals (CI) for each group. (b) Box plots showing group dispersions with Bray–Curtis distance matrix (betadisper analyses) of adults and nestlings from cavity and non‐cavity‐nester species.

Nest type was a good predictor of beta diversity indices for gland skin and secretion in both adults and nestlings (*F* > 1.33, *p* < 0.049, except for the weighted and unweighted UniFrac distance matrices of gland skin of adults, *F* < 1.03, *p* > 0.340; and weighted UniFrac of nestlings' secretion, *F* = 2.38, *p* = 0.068; Figure 3 and ESM Table S7). This pattern remained for most indices after controlling for host phylogeny (ESM Table S9). The proportion of variance of the bacterial community of the secretion explained by nest type seems to be larger in the UniFrac metrics of adults (unweighted *R* ^2^ = 0.03, weighted *R* ^2^ = 0.05) than in nestlings (unweighted *R* ^2^ = 0.01, weighted *R* ^2^ = 0.01, ESM Table S7). However, these *R* ^2^ were estimated for different datasets and should therefore be interpreted with caution. Moreover, pairwise comparisons of both bacterial communities (uropygial gland skin and secretion) in dispersion tests (average distance to spatial median) showed that distances to spatial median estimated for adults and nestlings of cavity‐nesting species were higher than those estimated for open‐cup‐nesting adults (*p* < 0.010, Figure 3b).

### 3.5 Is there a correlation (connectivity) between gland skin and secretion microbiotas?

**FIGURE 4:**
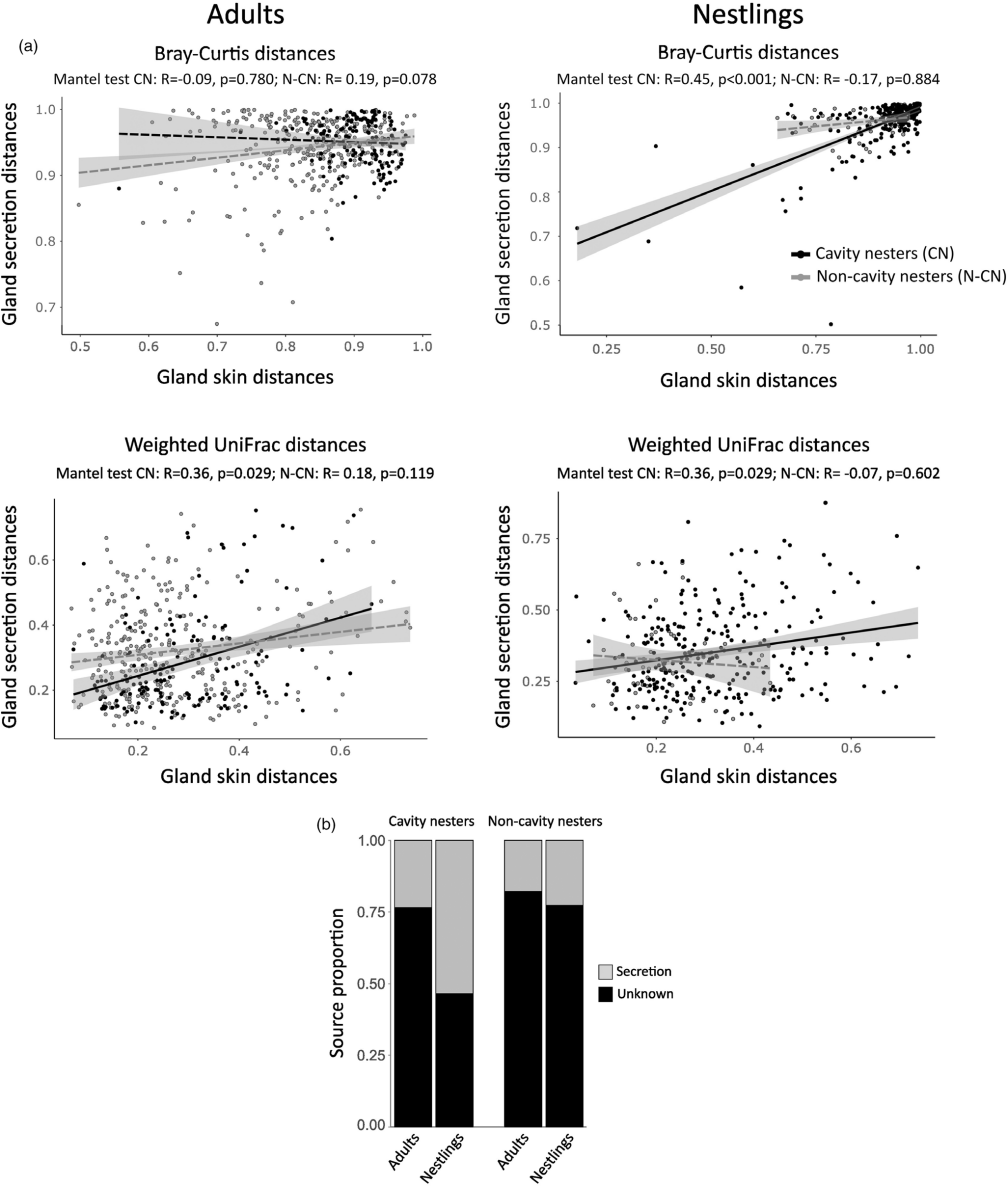
Bacterial community of the uropygial secretion and skin is associated with each other in cavity nesters. (a) Linear regression between distance matrices (Bray–Curtis and weighted UniFrac) of bacterial communities of the uropygial gland secretion and uropygial skin of adults (left) and nestlings (right) of cavity (CN) and open‐cup nesters (N‐CN). Solid lines indicate significant trends, while dashed lines indicate non‐significant trends. (b) Results of source‐tracking analyses showing the proportions of the uropygial gland skin microbiota that originated from the microbiota of the uropygial gland secretion or unknown sources.

In the subset of individuals with both successfully sequenced gland skin and secretion microbiomes (*N* = 80), we obtained a total of 12,005 ASVs (ESM Table S10). Mantel tests revealed that bacterial communities were significantly correlated for both adults and nestlings at the ASV level for all distance matrices (*R* > 0.16, *p* < 0.038; ESM Table S11), except for unweighted UniFrac distances (*R* < 0.04, *p* > 0.299; ESM Table S11). When considering only cavity‐nesting species, in nestling samples, the correlation between the bacterial communities was significant at the ASV and genus levels for Bray–Curtis and Jaccard distances, whereas the expected correlation in adult samples was instead detected for unweighted and weighted UniFrac distance matrices (Figure 4a, ESM Table S11). In contrast, for open‐cup nesters, we did not find any association between bacterial communities in adults or nestlings (Figure 4a, ESM, Table S11), even after controlling for the bird phylogeny (ESM Table S11). Consistent with this, effect sizes of the associations between the two communities were significantly larger in cavity than in open‐cup nesters. This was observed for Bray–Curtis (*R* 95% CI cavity nesters = 0.33–0.56; open‐cup nesters = (−0.25)–0.02) and Jaccard (*R* 95% CI cavity nesters = 0.32–0.59; open‐cup nesters = (−0.31)–0.03) distance matrices at the ASV level for nestlings, and for unweighted UniFrac distance matrix at the genus level in adults (*R* 95% CI cavity nesters = 0.12–0.43; open‐cup nesters = (−0.09)–0.09). See ESM Table S11 for compared effect size (*R*) values.

### 3.6 Does the bacterial community of the gland skin derive from gland secretions, especially in cavity‐nesting species and nestlings?

Source‐tracking analyses revealed that the proportion of the gland skin microbiota originating from the gland secretion differed across species for nestlings (*F* = 3.40, *p* = 0.021, ESM Table S12) but not for adults (*F* = 1.84, *p* = 0.104, ESM Table S12). These analyses revealed that cavity‐nester nestlings had more bacteria derived from the uropygial gland secretion than open‐cup nestlings (*F* = 7.48, *p* = 0.013, ESM Table S12, Figure 4b, Figure S3).

## 4 DISCUSSION

Here we explored multiple hypotheses related to the possible role of species identity, nest type (cavity vs. open‐cup nesting), and age in shaping the uropygial gland secretion and skin microbiomes of adults and nestlings of 26 European bird species. Supporting our first hypothesis, we detected larger glands in cavity‐nester species, suggesting that these species face higher pathogen pressures and might preen more often than open‐cup nesters (Galván & Sanz, 2006; Jacob et al., 2014; Magallanes et al., 2016; Møller et al., 2010; Moreno‐Rueda, 2010; Soler et al., 2012). Moreover, consistent with our second hypothesis, we found species‐specific secretion and gland skin microbiomes, similar to what has previously been found for feather microbiomes (Javůrková et al., 2019; Labrador et al., 2020). Variation in nest environment between species (Hanssell, 2000) affect eggshell bacterial communities (Peralta‐Sánchez et al., 2012; West et al., 2015), and this may in part cause the detected interspecific differences in uropygial bacterial communities. The diversity and composition of our bacterial communities were further affected by host age, as also found in the skin microbiota of zebra finches (*Taeniopygia guttata*, Engel et al., 2020), but not in the microbiota of the uropygial gland skin, feathers and bill of the cavity‐nesting African hornbills (Fam. Bucerotidae, Barón et al., 2024). Moreover, the effects of age varied among species, which may be explained by species‐specific characteristics influencing bacterial communities (i.e. foraging behaviour, social interactions, preening and other sanitation behaviours and nest material used, Bodawatta et al., 2022; Grieves et al., 2023; Martínez‐Renau et al., 2024; Peralta‐Sánchez et al., 2012). The effect of age on alpha diversity was significantly larger for the skin microbiota, which is more in contact with the nest environment than the secretion microbiota. The effect of age may be caused by nestlings spending more time in the nests than adults.

In accordance with our hypothesis for re‐used cavity nests, we found some evidence of cavity nesters harbouring compositionally different and more heterogeneous bacterial communities than open‐cup nesters. However, diversity was only higher in the secretion of cavity‐nester adults. Higher diversity may be driven by increased risks of pathogenic infection in cavity nesters (Peralta‐Sánchez et al., 2012). It could also suggest a direct effect of the surrounding nest environment determining uropygial skin and secretion microbiotas. While we cannot evaluate this directly, the stronger effect of nest type on secretions in adults than nestlings might support that nest‐type‐associated pathogen risks could influence microbiomes more than contact with the nest environment. Thus, consistent with findings by Peralta‐Sánchez et al. (2012), the nest environment does not directly explain the detected effects of nest type. However, it is worth noting that the detected effects of nest type could be confounded by sampling location or phylogeny. Avian communities of the two studied areas differ in the proportion of cavity vs. open‐nester species. We control for these sources of variation statistically, and although sampling location and nest type covary, we detected statistically significant effects of the latter. This suggests that nest type is an important predictor of gland skin and secretion microbiota composition.

Our results also suggest that the uropygial gland is larger in cavity nesters than in open‐cup nesters, which implies that cavity nesters preen more often than open‐cup nesters (Giraudeau et al., 2017; Leclaire et al., 2014). Consistent with this, we observed stronger associations between gland secretions and skin microbiomes in cavity nesters, and a larger proportion of the skin microbiota originating from the uropygial gland in cavity‐nester nestlings. Potential higher risk of pathogenic infection that species breeding in cavities experience (López‐Rull & Macías‐García, 2015; Peralta‐Sánchez et al., 2012) could explain the microbiome differences and development of larger defensive uropygial glands (Vincze et al., 2013). Larger glands may facilitate higher preening frequencies, which can lead to increased transfer of bacteria from the uropygial gland to the bird integuments (i.e. skin) and, thus, the detected stronger associations.

Differential antimicrobial properties of the uropygial gland secretions of cavity‐nesting species could also explain the effects of nest type on the microbiomes of the uropygial gland secretion and skin; simply because antimicrobial chemicals influence the diversity and composition of bacterial communities (Riley & Wertz, 2002; Widder et al., 2016). Cavity nesters seem to have richer, compositionally different and more heterogeneous gland secretion and skin microbiomes than open‐cup nesters. This may be a consequence of differences in chemical or antimicrobial properties of the secretion (Haribal et al., 2009; Jacob et al., 2018; Jacob & Ziswiler, 1982) and the antimicrobial production of bacteria growing in these locations (Riley & Wertz, 2002). In a previous study, Martínez‐Renau et al. (2022) isolated more bacterial strains with antimicrobial capabilities from the uropygial skin of cavity nesters than from that of open‐cup nesters, which might therefore explain the detected differences associated with nest type. Thus, it is possible that cavity nesters would potentially need more diverse and variable symbiotic communities to counteract pathogens in nest environments where they are more prevalent; a possibility worth exploring in the future.

The strength of microbiota connection appears to be host‐age‐dependent, as we only observed differences in microbiome connectivity between nestlings with varying nesting type. Adults frequently abandon the nests, where bacteria from other sources could colonise their uropygial skin, potentially masking the impact of uropygial secretion microbiota on the uropygial skin microbiota. Alternatively, since nestlings are more vulnerable to pathogens than adults (Palacios et al., 2009; Stambaugh et al., 2011), antimicrobial‐producing symbionts from the uropygial gland reaching bird integuments should be particularly important for juveniles. However, this contradicts the stronger inhibition and antimicrobial activity ranges found in adult bacterial symbionts compared to those of nestlings (Martínez‐Renau et al., 2022). Another possibility explaining the detected age differences in connectivity is related to the fact that nestlings are not fully developed. Age‐related differences in morphology and/or physiology of organs would not only affect particularities of the established bacterial communities (Maraci et al., 2022; van Dongen et al., 2013; Zhou et al., 2020), but also their connectivity. For instance, the chemical composition or the microbiota of the secretion may not be fully matured in nestlings and, because their associated microbiota have lower antimicrobial activity (Martínez‐Renau et al., 2022), they may need to preen more frequently to maintain feather or skin protection.

There may also be non‐adaptive explanations for our connectivity results. Cavity nests are partially sheltered from environmental impacts and thus more stable (Riquelme et al., 2015) and isolated from airborne microbes (Godard et al., 2007). This would predict a stronger influence of the secretion microbiota on the skin bacterial community in cavity nesters and nestlings. However, these microbiological conditions do not apply to cavities with previous breeding activities, where remains and debris from previous reproduction attempts increase the abundance and diversity of microbes, and therefore influence the composition of the nest bacterial environments and their occupants (Diaz‐Lora et al., 2019; Peralta‐Sánchez et al., 2012; Xin et al., 2023; Zabłotni et al., 2023). This could lead to fast colonisation of the skin and dilute effects of the gland secretion. Since our results show associations for nestlings of species nesting in re‐used cavities, it is unlikely that nest environment itself (i.e. climatic and microbiological conditions of cavity nests) explains the observed influence of age and nest type in the connectivity. Higher risk of pathogenic infection may be a more likely explanation, but future research is needed to better elucidate the drivers of connectivity between these bacterial communities.

In summary, we found some evidence suggesting that the nest type used by birds is an important predictor of the diversity and composition of the symbiotic bacterial communities of their uropygial gland skin, and the secretion produced in it, as well as the connectivity between both bacterial communities. Our findings provide new insights into the potential role of pathogen pressures determining the diversity and composition of exocrine gland and integument bacterial communities.

## AUTHOR CONTRIBUTIONS

Juan José Soler and Manuel Martín‐Vivaldi conceived and designed the experiments. Ester Martínez‐Renau, Manuel Martín‐Vivaldi, María Dolores Barón, Cristina Ruiz‐Castellano and Juan José Soler did the fieldwork. Antonio M. Martín‐Platero and Manuel Martínez‐Bueno performed the laboratory work. Ester Martínez‐Renau, Juan José Soler, Kasun H. Bodawatta and Knud A. Jønsson analysed the data. Ester Martínez‐Renau wrote the first version of the manuscript under the supervision of Juan José Soler, Antonio M. Martín‐Platero, Kasun H. Bodawatta and Michael Poulsen. All authors contributed substantially to the final version of the manuscript and gave final approval for publication.

## CONFLICT OF INTEREST STATEMENT

The authors declare no conflict of interest.

## ETHICS STATEMENT

This study was reviewed and approved by the Environmental Department of the Regional Government of Andalucía, Spain (Consejería de Agricultura, ganaderia, Pesca y Desarrollo Sostenible, reference DGPAG/ SA/SIS).

## Supporting information


**Figure S1.** Bird phylogenetic tree built using three mitochondrial genes: cytochrome b (cyt b), NADH dehydrogenase subunit 2 (ND2), and cytochrome oxidase I (COI).
**Figure S2.** Cavity and open‐cup nester species show different patterns in the abundance distribution of the most common bacterial genera. Heatmap showing the relative abundance of the 24 most frequently detected bacterial genera (phylum is given in parentheses) and their relative abundances for each of the 26 bird species. For each of the four groups, the 12 most abundant bacterial genera were identified, and the combined set of these genera (24) was used to generate the heatmap. Taxonomy of the bird species is given on the left. Age class and nest type are represented in pictograms, and sample sizes are given for both age classes. Species considered in the source‐tracking analyses are marked with *, and grey bars represent missing data.
**Figure S3.** Results of the source‐tracking analyses showing the proportions of the microbiota of the uropygial gland skin that is originated from the microbiota of the uropygial gland secretion versus an unknown source for each species.
**Table S1.** Results of PERMANOVA analyses exploring the effects of species ID, age class and nest type on beta diversity (distance matrices constructed with Aitchison and Euclidean distances computed on the CLR‐ and PhILR‐transformed ASV tables, respectively). Species ID and age class effects were explored together in models including both variables and the nest identity nested within the interaction of species ID and age class. The interaction between species ID and age was calculated in separate models that also included the main effects. The effect of nest type was explored in nested models that included nest type, species ID nested within nest type and nest identity nested within species ID (in turn nested within nest type). Values in bold are those with associated *p*‐value lower than 0.05.
**Table S2.** Results of Linear Models exploring the effects of species identity, age class and nest type on the Box‐Cox transformed alpha diversity indexes. These models were performed removing all the samples from individual fledglings from the data set. Species ID and age class effects were explored together in models including both variables, while the interaction was calculated in separate models that also included the main effects. The effect of nest type was explored in a nested LM model that included nest type and species ID nested within nest type. Nest identity was added as random factor. Values in bold are those with associated *p*‐value lower than 0.05. LRT is reported for random variables instead of the *F* statistic.
**Table S3.** Results of PERMANOVAs exploring the effects of species identity, age class and nest type on the beta diversity (Bray–Curtis, Jaccard, weighted UniFrac and unweighted UniFrac distance matrices). These models were performed removing all the samples from individual fledglings from the data set. Species ID and age class effects were explored together in models including both variables and using nest ID as the strata parameter to restrict permutations. The interaction between species ID and age was calculated in separate models that also included the main effects. The effect of nest type was explored in nested models that included nest type, species ID nested within nest type and (only for nestling models) nest identity nested within species ID (in turn nested within nest type). Values in bold are those with associated p‐value lower than 0.05.
**Table S6.** Results of linear models (LMs) exploring the effects of species identity (ID), age class and nest type on Box‐Cox transformed alpha diversity variables (Chao1, Shannon index and PD). Species identity and age class effects were explored together in models including both variables and the sampling locality, while the interaction was calculated in separate models that also included the main effects. The effect of nest type was explored in a nested LM model that included nest type, species ID nested within nest type and the sampling locality. In models including nestling samples, nest identity was added as random factor. Values in bold are those with associated *p*‐value lower than 0.05. Cohen's *d* of age and nest type are included as a metric of effect size. LRT is reported for random variables instead of the *F* statistic.
**Table S7.** Results of PERMANOVA analyses exploring the effects of species identity (ID), age class and nest type on beta diversity (Bray–Curtis, Jaccard, weighted UniFrac and unweighted UniFrac distance matrices). Species ID and age class effects were explored together in models including both variables and sampling locality, using nest as the strata parameter to restrict permutations. The interaction between species ID and age was calculated in separate models that also included the main effects. The effect of nest type was explored in nested models that included nest type, species ID nested within nest type, sampling locality and (only for nestling models) nest identity nested within species ID (in turn nested within nest type). Values in bold are those with associated *p*‐value lower than 0.05 and *F*.
**Table S8.** Results of MCMCglmm models with nest type as a fixed factor, sampling locality and the bird phylogeny as a random factor, and the Box‐Cox transformed alpha diversity variables (Chao1, Shannon index and PD) as dependent variables. The species *Columba livia* and *Passer montanus* were removed from the phylogeny controlled analyses. CI refers here to Credible Interval for the nest type. Values in bold are those with associated *p*‐values lower than 0.05.
**Table S9.** Results of Mantel tests of effects of nest type on beta diversity (Bray–Curtis, Jaccard and weighted and unweighted UniFrac distance matrices) of the bacterial communities of secretion and skin of adult and nestlings after controlling for the bird phylogeny. Nest ID was included in the models of nestlings to control for the non‐independence of the data from siblings. The species *Columba livia* and *Passer montanus* were removed from the phylogeny controlled analyses. Values in bold are those with associated *p*‐values lower than 0.05.
**Table S11.** Results from Mantel tests exploring the correlations between microbiomes of the uropygial gland skin and uropygial gland secretion in adults and nestlings. We also show the results after controlling for the bird phylogeny. The distance matrix from the uropygial skin was defined as the dependent matrix, while the secretion distance matrix was defined as the independent matrix. A bird phylogeny distance matrix was included in the phylogenetically controlled analyses. The species *Columba livia* and *Passer montanus* were removed from the phylogeny controlled analyses. Results are shown for the entire set of samples, as well as subsets for cavity and non‐cavity nesters, at both the ASV and genus level. Bray–Curtis, Jaccard, weighted UniFrac and unweighted UniFrac were used to build distance matrices. The number of permutations was set to 10,000. Values in bold are those with associated alpha values lower than 0.05.
**Table S12.** Results of general linear models using source‐tracking (FEAST) output. The proportion of the gland‐skin bacterial community originated from the gland secretion was used as dependent variable. We explored the effects of age class, species identity (ID) and nest type in two different models. The first model included the species ID and age class as fixed factors, while the interaction between them was analysed in separate models including also the main effects. The effect of nest type was explored separately for adults and nestlings, including the nest type, sampling locality and the species ID nested within nest type as independent variables. Values in bold are those with associated alpha value lower than 0.05. For random factors, LRT is reported instead of the *F* statistic.

**Table S4.** Rarefied ASV table showing the abundance of the ASVs for each of the uropygial gland skin samples. ASV taxonomy and sample metadata are also shown (separate excel file).
**Table S5.** Rarefied ASV table showing the abundance of the ASVs for each of the uropygial gland secretion samples. ASV taxonomy and sample metadata are also shown (separate excel file).
**Table S10.** Rarefied ASV table showing the abundance of the ASVs for each of the uropygial gland skin and secretion samples used in the source‐tracking analyses. ASV taxonomy and sample metadata are also shown (separate excel file).

## Data Availability

Amplicon sequences have been uploaded to the SRA archive in GenBank (accession no.: PRJNA1248315). Data and R code used in this paper can be found in CSIC Institutional Repository https://doi.org/10.20350/digitalCSIC/18328 (Martínez‐Renau et al., 2026).
