# Supplementary material for: Environmental factors associated with nesting habits and age shape the composition and connection between skin and uropygial gland microbiomes of birds
Source: J Anim Ecol. 2026 Jun 30;95(8):1435–52. doi: 10.1111/1365-2656.70304 (PMC13439605; doi:10.1111/1365-2656.70304)
Supplement: Supplementary file 1 [file JANE-95-1435-s002.docx]

**Nesting habit-associated pathogen pressures influence the composition and connection between skin and uropygial gland microbiomes of birds**

**Electronical Supplementary Material**

Bird phylogenetic tree construction

We generated a phylogenetic tree of the avian hosts using three mitochondrial genes (Cytochrome b [cyt b], NADH dehydrogenase subunit 2 [ND2] and cytochrome oxidase subunit I [COI]) sourced from GenBank and built individual gene alignments using MAFFT (Katoh, Misawa, Kuma & Miyata, 2002) in SEAVIEW (Gouy, Guindon & Gascuel, 2010). We then analysed individual gene partitions in BEAST v1.8.4 (Drummond, Suchard, Xie & Rambaut, 2012) as an initial way to check the quality of the sequence data. Subsequently, we generated a concatenated dataset (2,905 base pairs) partitioned by genes in BEAST v1.8.4 (Drummond et al., 2012) using the GTR + I + G nucleotide substitution model as determined by jModelTest (Guindon & Gascuel, 2003; Darriba, Taboada, Doallo & Posada, 2012). We used a relaxed uncorrelated lognormal distribution for the molecular clock model and assumed a birth-death speciation process as a tree prior. The Markov chain Monte Carlo (MCMC) algorithm was run several times for 100 million iterations with trees sampled every 10,000th generation. Convergence of individual runs was assessed using Tracer 1.6 (Rambaut, Suchard, Xie & Drummond, 2014) ensuring all ESS > 200 and graphically estimating an appropriate burn-in. TreeAnnotator 1.8.2 (Rambaut & Drummond, 2015) was used to summarize a single maximum clade credibility (MCC) tree using mean node heights. We constrained the relationships of our tree by locking avian orders according to Jarvis et al. (2014) and Prum et al. (2015) and by locking passerine bird families according to Oliveros et al. (2019). To obtain absolute dates, we applied 1) a rate of 0.0145 substitutions per site per lineage (2.9%) per Myr to our ND2 data, 2) a rate of 0.007 substitutions per site per lineage (1.4%) per Myr to our cyt-b data, and 3) a rate of 0.008 substitutions per site per lineage (1.6%) per Myr to our COI data following Lerner, Meyer, James, Hofreiter and Fleischer (2011).

**Figure S1**. Bird phylogenetic tree built using three mitochondrial genes: cytochrome b (cyt b), NADH dehydrogenase subunit 2 (ND2), and cytochrome oxidase I (COI).

**Figure S2**. **Cavity and open-cup nester species show different patterns in the abundance distribution of the most common bacterial genera**. Heatmap showing the relative abundance of the 24 most frequently detected bacterial genera (phylum is given in parentheses) and their relative abundances for each of the 26 bird species. For each of the four groups, the 12 most abundant bacterial genera were identified, and the combined set of these genera (24) was used to generate the heatmap. Taxonomy of the bird species is given on the left. Age class and nest type are represented in pictograms, and sample sizes are given for both age classes. Species considered in the source tracking analyses are marked with *, and grey bars represent missing data.

**Figure S3.** Results of the source-tracking analyses showing the proportions of the microbiota of the uropygial gland skin that is originated from the microbiota of the uropygial gland secretion versus an unknown source for each species.

**Table S1.** Results of PERMANOVA analyses exploring the effects of species ID, age class and nest type on beta diversity (distance matrices constructed with Aitchison and Euclidean distances computed on the CLR- and PhILR-transformed ASV tables, respectively). Species ID and age class effects were explored together in models including both variables and the nest identity nested within the interaction of species ID and age class. The interaction between species ID and age was calculated in separate models that also included the main effects. The effect of nest type was explored in nested models that included nest type, species ID nested within nest type and nest identity nested within species ID (in turn nested within nest type). Values in bold are those with associated p-value lower than 0.05.

|  |  |  | **CLR** | | | **PhILR** | | |
| --- | --- | --- | --- | --- | --- | --- | --- | --- |
|  |  |  | Pseudo-F | *df* | p | Pseudo-F | *df* | p |
| **Effect of age class** | | |  |  |  |  |  |  |
|  | *Gland skin* | |  |  |  |  |  |  |
|  |  | Species | **5.85** | **7, 251** | **<0.001** | **9.78** | **7, 251** | **<0.001** |
|  |  | Age | **7.47** | **1, 251** | **<0.001** | **8.34** | **1, 251** | **<0.001** |
|  |  | Locality | **4.91** | **1, 251** | **<0.001** | **10.66** | **1, 251** | **<0.001** |
|  |  | Species*Age | **2.26** | **7, 244** | **<0.001** | **2.59** | **7, 244** | **<0.001** |
|  | *Gland secretion* | |  |  |  |  |  |  |
|  |  | Species | **2.76** | **6, 188** | **<0.001** | **2.95** | **6, 188** | **0.005** |
|  |  | Age | **1.89** | **1, 188** | **<0.001** | **1.76** | **1, 188** | **0.008** |
|  |  | Locality | **1.89** | **1 , 188** | **<0.001** | **2.12** | **1, 188** | **0.003** |
|  |  | Species*Age | **1.50** | **6, 182** | **<0.001** | **1.45** | **6, 182** | **<0.001** |
| **Effect of nest type** | | |  |  |  |  |  |  |
| Adults | *Gland skin* | |  |  |  |  |  |  |
|  |  | Nest type | 3.41 | 1, 107 | 0.719 | 0.39 | 1, 107 | 0.892 |
|  |  | Species (Nest type) | **1.74** | **11, 107** | **<0.001** | **4.14** | **11, 107** | **<0.001** |
|  |  | Locality | **3.41** | **1, 107** | **<0.001** | **17.34** | **1, 107** | **<0.001** |
|  | *Gland secretion* | |  |  |  |  |  |  |
|  |  | Nest type | 1.14 | 1, 124 | 0.154 | 1.10 | 1, 124 | 0.254 |
|  |  | Species (Nest type) | **1.20** | **14, 124** | **<0.001** | **1.42** | **14, 124** | **<0.001** |
|  |  | Locality | **2.69** | **1, 124** | **<0.001** | **3.35** | **1, 124** | **<0.001** |
| Nestlings | *Gland skin* | |  |  |  |  |  |  |
|  |  | Nest type | **3.75** | **1, 95** | **<0.001** | **6.81** | **1, 95** | **<0.001** |
|  |  | Species (Nest type) | **8.15** | **12, 95** | **<0.001** | **13.76** | **12, 95** | **<0.001** |
|  |  | Locality | **3.40** | **1, 95** | **<0.001** | **6.85** | **1, 95** | **<0.001** |
|  |  | Nest (Species(Nest type)) | **1.84** | **108, 95** | **<0.001** | **1.98** | **108, 95** | **<0.001** |
|  | *Gland secretion* | |  |  |  |  |  |  |
|  |  | Nest type | **1.62** | **1, 114** | **0.001** | 1.18 | 1, 114 | 0.136 |
|  |  | Species (Nest type) | **2.98** | **11, 114** | **<0.001** | **3.09** | **11, 114** | **<0.001** |
|  |  | Locality | **1..26** | **1, 114** | **0.068** | **1.34** | **1, 114** | **0.058** |
|  |  | Nest (Species(Nest type)) | **1.31** | **83, 114** | **<0.001** | **1.35** | **83, 114** | **<0.001** |

|  |  |  | **Chao1** | | | | **Shannon index** | | | | **PD** | | | |
| --- | --- | --- | --- | --- | --- | --- | --- | --- | --- | --- | --- | --- | --- | --- |
|  |  |  | F/LRT | *df* | p | F/LRT | | *df* | p | F/LRT | | *df* | p |  |
| **Effect of age class** | | |  |  |  |  | |  |  |  | |  |  |  |
|  | *Gland skin* | |  |  |  |  | |  |  |  | |  |  |  |
|  |  | Species (f) | **9.94** | **7, 120.1** | **<0.001** | **3.99** | | **7, 151.9** | **<0.001** | **3.49** | | **7, 121.8** | **0.002** |  |
|  |  | Age (f) | **126.87** | **1, 190.9** | **<0.001** | **19.76** | | **1, 225.7** | **<0.001** | 0.13 | | 1, 200.6 | 0.724 |  |
|  |  | Species*Age (f) | 3.04 | 6, 191.5 | 0.007 | **7.32** | | **6, 212.0** | **<0.001** | **5.01** | | **6, 200.4** | **<0.001** |  |
|  |  | Nest (rnd) | **19.37** | **1** | **<0.001** | **12.00** | | **1** | **0.001** | **14.83** | | **1** | **<0.001** |  |
|  | *Gland secretion* | |  |  |  |  | |  |  |  | |  |  |  |
|  |  | Species (f) | **4.92** | **6, 88.4** | **<0.001** | **4.12** | | **6, 94.1** | **0.001** | **4.56** | | **6, 84.7** | **<0.001** |  |
|  |  | Age (f) | **12.43** | **1, 175.7** | **<0.001** | 0.97 | | 1, 174.7 | 0.326 | 3.83 | | 1, 174.8 | 0.052 |  |
|  |  | Species*Age (f) | **3.49** | **5, 132.9** | **0.005** | **6.10** | | **5, 138.4** | **<0.001** | **6.48** | | **5, 131.0** | **<0.001** |  |
|  |  | Nest (rnd) | **38.45** | **1** | **<0.001** | **34.55** | | **1** | **<0.001** | **35.83** | | **1** | **<0.001** |  |
| **Effect of nest type** | | |  |  |  |  | |  |  |  | |  |  |  |
| Nestlings | *Gland skin* | |  |  |  |  | |  |  |  | |  |  |  |
|  |  | Nest type (f) | 1.61 | 1, 121.1 | 0.206 | **5.24** | | **1, 122.5** | **0.024** | **4.27** | | **1, 124.5** | **0.041** |  |
|  |  | Species (nest type) (f) | **17.02** | **11, 94.4** | **<0.001** | **4.74** | | **11, 98.4** | **<0.001** | **10.69** | | **11, 93.3** | **<0.001** |  |
|  |  | Nest (Species(Nest type)) (rnd) | **15.97** | **1** | **<0.001** | **20.18** | | **1** | **<0.001** | **8.99** | | **1** | **0.003** |  |
|  | *Gland secretion* | |  |  |  |  | |  |  |  | |  |  |  |
|  |  | Nest type (f) | 0.55 | 1, 110.2 | 0.459 | <0.01 | | 1, 118.4 | 0.946 | 0.14 | | 1, 118.6 | 0.708 |  |
|  |  | Species (nest type) (f) | **2.71** | **10, 57.0** | **0.009** | **2.54** | | **10, 70.7** | **0.011** | **2.44** | | **10, 55.4** | **0.017** |  |
|  |  | Nest (Species(Nest type)) (rnd) | **10.96** | **1** | **<0.001** | **21.28** | | **1** | **<0.001** | **6.89** | | **1** | **0.009** |  |

**Table S2.** Results of Linear Models exploring the effects of species identity, age class and nest type on the Box-Cox transformed alpha diversity indexes. These models were performed removing all the samples from individual fledglings from the data set. Species ID and age class effects were explored together in models including both variables, while the interaction was calculated in separate models that also included the main effects. The effect of nest type was explored in a nested LM model that included nest type and species ID nested within nest type. Nest identity was added as random factor. Values in bold are those with associated p-value lower than 0.05. LRT is reported for random variables instead of the F statistic.

|  |  |  | **Bray-Curtis** | | | **Jaccard** | | | **Unweighted UniFrac** | | | **Weighted UniFrac** | | | |  |
| --- | --- | --- | --- | --- | --- | --- | --- | --- | --- | --- | --- | --- | --- | --- | --- | --- |
|  |  |  | F | *df* | p | F | *df* | p | F | *df* | p | | F | *df* | p | |
| **Effect of age class** | | |  |  |  |  |  |  |  |  |  | |  |  |  | |
|  | *Gland skin* | |  |  |  |  |  |  |  |  |  | |  |  |  | |
|  |  | Species (f) | **10.40** | **7, 242** | **<0.001** | **6.27** | **7, 242** | **<0.001** | **8.57** | **7, 242** | **<0.001** | | 33.80 | 7, 242 | 0.684 | |
|  |  | Age (f) | **8.49** | **1, 242** | **<0.001** | **5.36** | **1, 242** | **<0.001** | **11.1** | **1, 242** | **<0.001** | | 12.5 | 1, 242 | 0.912 | |
|  |  | Species*Age (f) | **2.97** | **6, 242** | **<0.001** | **2.17** | **6, 242** | **<0.001** | **3.65** | **6, 242** | **<0.001** | | **8.25** | **6, 242** | **<0.001** | |
|  | *Gland secretion* | |  |  |  |  |  |  |  |  |  | |  |  |  | |
|  |  | Species (f) | **3.77** | **6, 176** | **<0.001** | **2.59** | **6, 176** | **0.002** | **5.23** | **6, 176** | **<0.001** | | **6.20** | **6, 176** | **<0.001** | |
|  |  | Age (f) | **3.74** | **1, 176** | **<0.001** | **2.52** | **1, 176** | **<0.001** | **5.75** | **1, 176** | **<0.001** | | **8.23** | **1, 176** | **<0.001** | |
|  |  | Species*Age (f) | **2.13** | **5, 176** | **<0.001** | **1.67** | **5, 176** | **<0.001** | **2.96** | **5, 176** | **<0.001** | | **2.95** | **5, 176** | **0.001** | |
| **Effect of nest type** | | |  |  |  |  |  |  |  |  |  | |  |  |  | |
| Nestlings | *Gland skin* | |  |  |  |  |  |  |  |  |  | |  |  |  | |
|  |  | Nest type (f) | **8.27** | **1, 95** | **<0.001** | **4.30** | **1, 95** | **<0.001** | **9.69** | **1, 95** | **<0.001** | | **23.28** | **1, 95** | **<0.001** | |
|  |  | Species (Nest type) (f) | **17.02** | **11, 95** | **<0.001** | **8.41** | **11, 95** | **<0.001** | **12.81** | **11, 95** | **<0.001** | | **29.01** | **11, 95** | **<0.001** | |
|  |  | Nest (Species(Nest type)) (f) | **3.37** | **100, 95** | **<0.001** | **2.50** | **100, 95** | **<0.001** | **1.74** | **100, 95** | **<0.001** | | **2.78** | **100, 95** | **<0.001** | |
|  | *Gland secretion* | |  |  |  |  |  |  |  |  |  | |  |  |  | |
|  |  | Nest type (f) | **2.89** | **1, 114** | **<0.001** | **1.99** | **1, 114** | **<0.001** | **2.58** | **1, 114** | **0.005** | | 2.12 | 1, 114 | 0.093 | |
|  |  | Species (Nest type) (f) | **5.38** | **10, 114** | **<0.001** | **3.28** | **10, 114** | **<0.001** | **5.67** | **10, 114** | **<0.001** | | **9.03** | **10, 114** | **<0.001** | |
|  |  | Nest (Species(Nest type)) (f) | **1.87** | **72, 114** | **<0.001** | **1.54** | **72, 114** | **<0.001** | **1.42** | **72, 114** | **<0.001** | | **1.91** | **72, 114** | **<0.001** | |

**Table S3.** Results of PERMANOVAs exploring the effects of species identity, age class and nest type on the beta diversity (Bray-Curtis, Jaccard, weighted UniFrac and unweighted UniFrac distance matrices). These models were performed removing all the samples from individual fledglings from the data set. Species ID and age class effects were explored together in models including both variables and using nest ID as the strata parameter to restrict permutations. The interaction between species ID and age was calculated in separate models that also included the main effects. The effect of nest type was explored in nested models that included nest type, species ID nested within nest type and (only for nestling models) nest identity nested within species ID (in turn nested within nest type). Values in bold are those with associated p-value lower than 0.05.

**Table S4:** Rarefied ASV table showing the abundance of the ASVs for each of the uropygial gland skin samples. ASV taxonomy and sample metadata are also shown (separate excel file).

**Table S5:** Rarefied ASV table showing the abundance of the ASVs for each of the uropygial gland secretion samples. ASV taxonomy and sample metadata are also shown (separate excel file).

**Table S6.** Results of Linear Models (LMs) exploring the effects of species identity (ID), age class and nest type on Box-Cox transformed alpha diversity variables (Chao1, Shannon index and PD). Species identity and age class effects were explored together in models including both variables and the sampling locality, while the interaction was calculated in separate models that also included the main effects. The effect of nest type was explored in a nested LM model that included nest type, species ID nested within nest type and the sampling locality. In models including nestling samples, nest identity was added as random factor. Values in bold are those with associated p-value lower than 0.05. Cohen’s d of age and nest type are included as a metric of effect size. LRT is reported for random variables instead of the F statistic.

|  |  |  | **Chao1** | | | | **Shannon index** | | | | **PD** | | | |  |
| --- | --- | --- | --- | --- | --- | --- | --- | --- | --- | --- | --- | --- | --- | --- | --- |
|  |  |  | F/LRT | *df* | p | Cohens’ d (95% CI) | F/LRT | *df* | p | Cohens’ d (95% CI) | F/LRT | *df* | p | Cohens’ d (95% CI) | |
| **Effect of age class** | | |  |  |  |  |  |  |  |  |  |  |  |  |  |
|  | *Gland skin* | |  |  |  |  |  |  |  |  |  |  |  |  |  |
|  |  | Species (f) | **5.07** | **7, 124.9** | **<0.001** |  | **4.45** | **7, 158.12** | **<0.001** |  | **3.32** | **7, 132.5** | **0.003** |  |  |
|  |  | Age (f) | **107.06** | **1, 215.4** | **<0.001** | **1.50 (1.19-1.83)** | **16.67** | **1, 233.5** | **<0.001** | **0.55 (0.27-0.84)** | 0.10 | 1, 218.7 | 0.753 | 0.04 ((-0.23)-0.33) |  |
|  |  | Locality (f) | **14.07** | **1, 178.5** | **<0.001** |  | **4.79** | **1, 220.6** | **0.013** |  | **20.5** | **1, 195.8** | **<0.001** |  |  |
|  |  | Species*Age (f) | **3.47** | **7, 202.5** | **0.002** |  | **6.85** | **7, 223.7** | **<0.001** |  | **3.54** | **7, 216.5** | **0.001** |  |  |
|  |  | Nest (rnd) | **17.07** | **1** | **<0.001** |  | **11.84** | **1** | **0.001** |  | **12.57** | **1** | **<0.001** |  |  |
|  | *Gland secretion* | |  |  |  |  |  |  |  |  |  |  |  |  |  |
|  |  | Species (f) | **2.44** | **6, 98.0** | **0.030** |  | 1.46 | 6, 106.0 | 0.200 |  | 1.27 | 6, 91.9 | 0.278 |  |  |
|  |  | Age (f) | **7.38** | **1, 188.0** | **0.007** | **0.41 (0.13-0.70)** | 1.81 | 1, 185.4 | 0.180 | 0.21 ((-0.07)-0.49) | 3.82 | 1, 187.9 | 0.052 | 0.29 (0.02-0.58) |  |
|  |  | Locality (f) | **14.98** | **1, 130.4** | **<0.001** |  | **8.88** | **148.0** | **0.003** |  | **15.61** | **1, 123.4** | **<0.001** |  |  |
|  |  | Species*Age (f) | 1.16 | 6, 141.6 | 0.332 |  | **3.82** | **6, 149.8** | **0.001** |  | **4.05** | **6, 139.2** | **<0.001** |  |  |
|  |  | Nest (rnd) | **35.77** | **1** | **<0.001** |  | **33.3** | **1** | **<0.001** |  | **30.89** | **1** | **<0.001** |  |  |
| **Effect of nest type** | | |  |  |  |  |  |  |  |  |  |  |  |  |  |
| Adults | *Gland skin* | |  |  |  |  |  |  |  |  |  |  |  |  |  |
|  |  | Nest type (f) | 0.91 | 1, 107 | 0.342 | 0.06 ((-0.29)-0.42) | 0.21 | 1, 107 | 0.650 | 0.26 ((-0.10)-0.62) | 0.15 | 1, 107 | 0.699 | 0.25 ((-0.11)-0.61) |  |
|  |  | Species (nest type) (f) | **2.89** | **11, 107** | **0.002** |  | **12.50** | **11, 107** | **<0.001** |  | **3.87** | **11, 107** | **<0.001** |  |  |
|  |  | Locality (f) | **9.27** | **1, 107** | **0.003** |  | **14.15** | **1, 107** | **<0.001** |  | **14.58** | **1, 107** | **<0.001** |  |  |
|  | *Gland secretion* | |  |  |  |  |  |  |  |  |  |  |  |  |  |
|  |  | Nest type (f) | **14.30** | **1, 124** | **<0.001** | **0.58 (0.24-0.92)** | **13.85** | **1, 124** | **<0.001** | **0.49 (0.15-0.82)** | **14.64** | **1, 124** | **<0.001** | **0.51 (0.17-0.85)** |  |
|  |  | Species (nest type) (f) | **3.42** | **14, 124** | **<0.001** |  | **3.08** | **14, 124** | **<0.001** |  | **3.03** | **14, 124** | **<0.001** |  |  |
|  |  | Locality (f) | **5.90** | **1, 124** | **0.017** |  | **5.65** | **1, 124** | **0.019** |  | **10.10** | **1, 124** | **0.002** |  |  |
| Nestlings | *Gland skin* | |  |  |  |  |  |  |  |  |  |  |  |  |  |
|  |  | Nest type (f) | 0.80 | 1, 146.5 | 0.373 | 0.05 ((-0.22)-0.32) | 2.72 | 1, 149.1 | 0.101 | 0.75 (0.48-1.04)) | 2.99 | 1, 155.9 | 0.086 | 0.59 (0.32-0.86) |  |
|  |  | Species (nest type) (f) | **15.84** | **12, 102.9** | **<0.001** |  | **4.56** | **12, 108.4** | **<0.001** |  | **10.18** | **12, 101.8** | **<0.001** |  |  |
|  |  | Locality (f) | **14.44** | **1, 149.4** | **<0.001** |  | **4.46** | **1, 151.7** | **0.036** |  | **6.65** | **1, 159.2** | **0.011** |  |  |
|  |  | Nest (Species(Nest type)) (rnd) | **15.9** | **1** | **<0.001** |  | **20.05** | **1** | **<0.001** |  | **8.94** | **1** | **0.003** |  |  |
|  | *Gland secretion* | |  |  |  |  |  |  |  |  |  |  |  |  |  |
|  |  | Nest type (f) | 0.03 | 1, 84.2 | 0.866 | 0.18 ((-0.09)-0.46) | <0.01 | 1, 99.1 | 0.966 | 0.09 ((-0.18)-0.37) | 0.36 | 1, 84.2 | 0.550 | 0.12 ((-0.15)-0.39) |  |
|  |  | Species (nest type) (f) | **2.55** | **11, 66.4** | **0.010** |  | **2.31** | **11, 83.2** | **0.016** |  | **2.32** | **11, 62.6** | **0.019** |  |  |
|  |  | Locality (f) | **5.75** | **1, 141.3** | **0.018** |  | 2.89 | 1, 148.9 | 0.091 |  | **6.21** | **1, 147.6** | **0.014** |  |  |
|  |  | Nest (Species(Nest type)) (rnd) | **10.79** | **1** | **0.001** |  | **16.94** | **1** | **<0.001** |  | **6.88** | **1** | **0.009** |  |  |

**Table S7.** Results of PERMANOVA analyses exploring the effects of species identity (ID), age class and nest type on beta diversity (Bray-Curtis, Jaccard, weighted UniFrac and unweighted UniFrac distance matrices). Species ID and age class effects were explored together in models including both variables and sampling locality, using nest as the strata parameter to restrict permutations. The interaction between species ID and age was calculated in separate models that also included the main effects. The effect of nest type was explored in nested models that included nest type, species ID nested within nest type, sampling locality and (only for nestling models) nest identity nested within species ID (in turn nested within nest type). Values in bold are those with associated p-value lower than 0.05 and F.

|  |  |  | **Bray-Curtis** | | | | **Jaccard** | | | | **Unweighted UniFrac** | | | | **Weighted UniFrac** | | | |
| --- | --- | --- | --- | --- | --- | --- | --- | --- | --- | --- | --- | --- | --- | --- | --- | --- | --- | --- |
|  |  |  | F | *df* | p | R^2^ | F | *df* | p | R^2^ | F | *df* | p | R^2^ | F | *df* | p | R^2^ |
| **Effect of age class** | | |  |  |  |  |  |  |  |  |  |  |  |  |  |  |  |  |
|  | *Gland skin* | |  |  |  |  |  |  |  |  |  |  |  |  |  |  |  |  |
|  |  | Species (f) | **9.70** | **7, 251** | **<0.001** | **0.20** | **5.89** | **7, 251** | **<0.001** | **0.14** | **8.23** | **7, 251** | **<0.001** | **0.17** | 29.23 | **7, 251** | 0.865 | 0.39 |
|  |  | Age (f) | **6.67** | **1, 251** | **<0.001** | **0.02** | **4.47** | **1, 251** | **<0.001** | **0.01** | **8.81** | **1, 251** | **<0.001** | **0.03** | 4.13 | **1, 251** | 0.510 | 0.01 |
|  |  | Locality (f) | **12.40** | **1, 251** | **<0.001** | **0.04** | **6.97** | **1, 251** | **<0.001** | **0.02** | **14.37** | **1, 251** | **<0.001** | **0.04** | 71.4 | 1, 251 | 0.774 | 0.13 |
|  |  | Species*Age (f) | **2.67** | **7, 244** | **<0.001** | **0.05** | **2.00** | **7, 244** | **<0.001** | **0.04** | **3.00** | **7, 244** | **<0.001** | **0.06** | **7.20** | **7, 244** | **<0.001** | **0.08** |
|  | *Gland secretion* | |  |  |  |  |  |  |  |  |  |  |  |  |  |  |  |  |
|  |  | Species (f) | **3.72** | **6, 188** | **<0.001** | **0.10** | **2.57** | **6, 188** | **<0.001** | **0.07** | **4.76** | **6, 188** | **<0.001** | **0.12** | **6.41** | **6, 188** | **<0.001** | **0.16** |
|  |  | Age (f) | **2.58** | **1, 188** | **<0.001** | **0.01** | **1.89** | **1, 188** | **<0.001** | **0.01** | **4.40** | **1, 188** | **<0.001** | **0.02** | **5.47** | **1, 188** | **<0.001** | **0.02** |
|  |  | Locality (f) | **3.65** | **1, 188** | **<0.001** | 0.02 | **2.41** | **1, 188** | **<0.001** | **0.01** | **11.86** | **1, 188** | **<0.001** | **0.05** | **3.58** | **1, 188** | **<0.001** | **0.01** |
|  |  | Species*Age (f) | **1.90** | **6, 182** | **<0.001** | **0.05** | **1.54** | **6, 182** | **<0.001** | **0.04** | **2.41** | **6, 182** | **<0.001** | **0.06** | **2.94** | **6, 182** | **<0.001** | **0.07** |
| **Effect of nest type** | | |  |  |  |  |  |  |  |  |  |  |  |  |  |  |  |  |
| Adults | *Gland skin* | |  |  |  |  |  |  |  |  |  |  |  |  |  |  |  |  |
|  |  | Nest type (f) | **1.57** | **1, 107** | **0.045** | **0.01** | **1.33** | **1, 107** | **0.049** | **0.01** | 1.03 | 1, 107 | 0.353 | 0.01 | 0.99 | 1, 107 | 0.340 | <0.01 |
|  |  | Species (nest type) (f) | **3.34** | **11, 107** | **<0.001** | **0.23** | **2.31** | **11, 107** | **<0.001** | **0.18** | **2.87** | **11, 107** | **<0.001** | **0.20** | **16.16** | **11, 107** | **<0.001** | **0.49** |
|  |  | Locality | **11.25** | **1, 107** | **<0.001** | **0.07** | **6.55** | **1, 107** | **<0.001** | **0.05** | **17.59** | **1, 107** | **<0.001** | **0.11** | **76.58** | **1, 107** | **<0.001** | **0.21** |
|  | *Gland secretion* | |  |  |  |  |  |  |  |  |  |  |  |  |  |  |  |  |
|  |  | Nest type (f) | **1.41** | **1, 124** | **0.005** | **0.01** | **1.21** | **1, 124** | **0.006** | **0.01** | **4.47** | **1, 124** | **<0.001** | **0.03** | **7.43** | **1, 124** | **<0.001** | **0.05** |
|  |  | Species (nest type) (f) | **1.29** | **14, 124** | **<0.001** | **0.12** | **1.16** | **14, 124** | **<0.001** | **0.11** | **1.76** | **14, 124** | **<0.001** | **0.14** | **1.45** | **14, 124** | **0.037** | **0.13** |
|  |  | Locality | **3.27** | **1, 124** | **<0.001** | **0.02** | **2.23** | **1, 124** | **<0.001** | **0.02** | **21.2** | **1, 124** | **<0.001** | **0.12** | **7.45** | **1, 124** | **<0.001** | **0.05** |
| Nestlings | *Gland skin* | |  |  |  |  |  |  |  |  |  |  |  |  |  |  |  |  |
|  |  | Nest type (f) | **7.54** | **1, 95** | **<0.001** | **0.01** | **4.00** | **1, 95** | **<0.001** | **0.01** | **8.12** | **1, 95** | **<0.001** | **0.02** | **24.20** | **1, 95** | **<0.001** | **0.03** |
|  |  | Species (nest type) (f) | **15.88** | **12, 95** | **<0.001** | **0.29** | **7.90** | **12, 95** | **<0.001** | **0.20** | **12.02** | **12, 95** | **<0.001** | **0.33** | **26.81** | **12, 95** | **<0.001** | **0.42** |
|  |  | Locality | **13.03** | **1,95** | **<0.001** | **0.02** | **6.20** | **1,95** | **<0.001** | **0.01** | **8.60** | **1,95** | **<0.001** | **0.02** | **37.63** | **1,95** | **<0.001** | **0.05** |
|  |  | Nest (Species(Nest type)) (f) | **3.29** | **108, 95** | **<0.001** | **0.54** | **2.45** | **108, 95** | **<0.001** | **0.57** | **1.73** | **108, 95** | **<0.001** | **0.42** | **2.66** | **108, 95** | **<0.001** | **0.37** |
|  | *Gland secretion* | |  |  |  |  |  |  |  |  |  |  |  |  |  |  |  |  |
|  |  | Nest type (f) | **2.75** | **1, 114** | **<0.001** | **0.01** | **1.92** | **1, 114** | **<0.001** | **0.01** | **2.35** | **1, 114** | **0.007** | **0.01** | 2.38 | 1, 114 | 0.068 | 0.01 |
|  |  | Species (nest type) (f) | **5.04** | **11, 114** | **<0.001** | **0.17** | **3.09** | **11, 114** | **<0.001** | **0.12** | **5.40** | **11, 114** | **<0.001** | **0.19** | **8.56** | **11, 114** | **<0.001** | **0.25** |
|  |  | Locality | **2.95** | **1, 114** | **<0.001** | **0.01** | **1.97** | **1, 114** | **<0.001** | **0.01** | **6.05** | **1, 114** | **<0.001** | **0.02** | **5.41** | **1, 114** | **0.003** | **0.01** |
|  |  | Nest (Species(Nest type)) (f) | **1.83** | **83, 114** | **<0.001** | **0.46** | **1.51** | **83, 114** | **<0.001** | **0.45** | **1.46** | **83, 114** | **<0.001** | **0.40** | **1.86** | **83, 114** | **<0.001** | **0.42** |

**Table S8.** Results of MCMCglmm models with nest type as a fixed factor, sampling locality and the bird phylogeny as a random factor, and the Box-Cox transformed alpha diversity variables (Chao1, Shannon index and PD) as dependent variables. The species *Columba livia* and *Passer montanus* were removed from the phylogeny controlled analyses. CI refers here to Credible Interval for the nest type. Values in bold are those with associated p-values lower than 0.05.

|  |  |  | **Mean** | **Lower 95% CI** | **Upper 95% CI** | **pMCMC** | **Autocorr** | **Heritability median** | **Heritability**  **Lower 95% CI** | **Heritability**  **Upper 95% CI** | **z score** |
| --- | --- | --- | --- | --- | --- | --- | --- | --- | --- | --- | --- |
| Gland skin | Adults | Chao1 | -0.340 | -1.926 | 1.240 | 0.648 | -0.019 | 0.130 | 0.000 | 0.464 | 1.372 |
|  |  | Shannon | 0.563 | -1.585 | 2.939 | 0.621 | -0.063 | 0.713 | 0.214 | 2.037 | 2.216 |
|  |  | PD | 0.186 | -0.456 | 0.880 | 0.594 | 0.033 | 0.046 | 0.005 | 0.150 | -0.107 |
|  | Nestlings | Chao1 | 0.209 | -1.561 | 1.634 | 0.758 | 0.005 | 0.774 | 0.598 | 0.910 | -1.275 |
|  |  | Shannon | 1.817 | 0.166 | 3.745 | 0.061 | 0.003 | 0.324 | 0.042 | 1.497 | 1.238 |
|  |  | PD | 0.428 | -0.126 | 0.993 | 0.137 | 0.052 | 0.056 | 0.017 | 0.225 | 0.641 |
| Gland secretion | Adults | Chao1 | -1.564 | -2.750 | -0.285 | **0.017** | 0.002 | 0.147 | 0.032 | 0.441 | -0.331 |
|  |  | Shannon | -1.663 | -3.146 | -0.388 | **0.019** | 0.020 | 0.132 | 0.001 | 0.392 | -0.187 |
|  |  | PD | -0.549 | -0.966 | -0.064 | **0.021** | -0.031 | 0.111 | 0.002 | 0.389 | 0.420 |
|  | Nestlings | Chao1 | 0.139 | -1.209 | 1.469 | 0.842 | -0.011 | 0.003 | 0.000 | 0.391 | -0.425 |
|  |  | Shannon | 0.540 | -0.784 | 2.202 | 0.480 | -0.027 | 0.001 | 0.000 | 0.297 | -0.648 |
|  |  | PD | 0.241 | -0.158 | 0.578 | 0.208 | -0.070 | 0.004 | 0.001 | 0.258 | -0.302 |

**Table S9.** Results of Mantel tests of effects of nest type on beta diversity (Bray Curtis, Jaccard, and weighted and unweighted UniFrac distance matrices) of the bacterial communities of secretion and skin of adult and nestlings after controlling for the bird phylogeny. Nest ID was included in the models of nestlings to control for the non-independence of the data from siblings. The species *Columba livia* and *Passer montanus* were removed from the phylogeny controlled analyses. Values in bold are those with associated p-values lower than 0.05.

|  |  |  | **Bray Curtis** | | **Jaccard** | | **Unweighted UniFrac** | | **Weighted UniFrac** | |
| --- | --- | --- | --- | --- | --- | --- | --- | --- | --- | --- |
|  |  |  | R | p | R | p | R | p | R | p |
| Adults | *Gland skin* | |  | |  | |  | |  | |
|  |  | Nest type | -0.001 | 0.780 | <0.001 | 0.875 | -0.017 | 0.051 | -0.009 | 0.501 |
|  |  | Locality | **0.018** | **0.002** | **0.011** | **0.006** | 0.034 | <0.001 | **0.024** | **0.036** |
|  |  | Phylogeny | **0.001** | **<0.001** | **<0.001** | **<0.001** | **<0.001** | **<0.001** | **0.002** | **<0.001** |
|  | *Gland secretion* | |  | |  | |  | |  | |
|  |  | Nest type | **0.006** | **0.002** | **<0.001** | **0.003** | **0.024** | **<0.001** | **0.020** | **0.001** |
|  |  | Locality | **0.012** | **<0.001** | **<0.001** | **<0.001** | **0.037** | **<0.001** | 0.005 | 0.167 |
|  |  | Phylogeny | **<0.001** | **<0.001** | **<0.001** | **<0.001** | **<0.001** | **0.039** | <0.001 | 0.204 |
| Nestlings | *Gland skin* | |  | |  | |  | |  | |
|  |  | Nest type | **0.021** | **0.003** | **0.01** | **<0.001** | 0.015 | 0.141 | 0.024 | 0.309 |
|  |  | Locality | **0.037** | **<0.001** | **0.026** | **<0.001** | 0.011 | 0.437 | 0.040 | 0.213 |
|  |  | Phylogeny | **<0.001** | **<0.001** | **<0.001** | **<0.001** | **<0.001** | **<0.001** | **<0.001** | **<0.001** |
|  |  | Nest ID | **0.084** | **<0.001** | **0.068** | **<0.001** | 0.006 | 0.649 | -0.011 | 0.735 |
|  | *Gland secretion* | |  | |  | |  | |  | |
|  |  | Nest type | 0.003 | 0.402 | 0.002 | 0.182 | **-0.023** | **0.017** | -0.029 | 0.105 |
|  |  | Locality | **0.026** | **<0.001** | **0.015** | **<0.001** | **0.084** | **<0.001** | **0.054** | **0.040** |
|  |  | Phylogeny | **<0.001** | **<0.001** | **<0.001** | **<0.001** | **<0.001** | **<0.001** | **<0.001** | **<0.001** |
|  |  | Nest ID | **0.019** | **<0.001** | **0.013** | **<0.001** | **0.002** | **0.660** | **0.032** | **0.005** |

**Table S10:** Rarefied ASV table showing the abundance of the ASVs for each of the uropygial gland skin and secretion samples used in the source-tracking analyses. ASV taxonomy and sample metadata are also shown (separate excel file).

**Table S11.** Results from Mantel tests exploring the correlations between microbiomes of the uropygial gland skin and uropygial gland secretion in adults and nestlings. We also show the results after controlling for the bird phylogeny. The distance matrix from the uropygial skin was defined as the dependent matrix, while the secretion distance matrix was defined as the independent matrix. A bird phylogeny distance matrix was included in the phylogenetically controlled analyses. The species *Columba livia* and *Passer montanus* were removed from the phylogeny controlled analyses. Results are shown for the entire set of samples, as well as subsets for cavity and non-cavity nesters, at both the ASV and genus level. Bray Curtis, Jaccard, weighted UniFrac and unweighted UniFrac were used to build distance matrices. The number of permutations was set to 10,000. Values in bold are those with associated alpha values lower than 0.05.

|  |  |  |  | | | | | | | | | | | | Phylogenetically controlled analyses | | | | | | | | |
| --- | --- | --- | --- | --- | --- | --- | --- | --- | --- | --- | --- | --- | --- | --- | --- | --- | --- | --- | --- | --- | --- | --- | --- |
|  |  |  | Gland skin microbiota | | | | | | | | | | | | Gland skin microbiota | | | | | | | | |
|  |  |  | **Bray Curtis** | | | **Jaccard** | | | **Unweighted UniFrac** | | | **Weighted UniFrac** | | | **Bray Curtis** | | **Jaccard** | | **Unweighted UniFrac** | | **Weighted UniFrac** | |  |
|  |  |  | R | p | CI | R | p | CI | R | p | CI | R | p | CI | R | p | R | p | R | p | R | p |  |
| **Adults** | All samples | **ASV-level** |  |  |  |  |  |  |  |  |  |  |  |  |  |  |  |  |  |  |  |  |  |
|  |  | Secretion microbiota | **0.16** | **0.023** | **0.08-0.25** | **0.18** | **0.024** | **0.08-0.25** | -0.01 | 0.546 | (-0.05)-0.03 | **0.25** | **0.012** | **0.12-0.33** | 0.24 | 0.087 | **0.30** | **0.042** | -0.01 | 0.949 | **0.22** | **0.016** |  |
|  |  | Phylogeny |  |  |  |  |  |  |  |  |  |  |  |  | **<0.01** | **0.128** | **<0.01** | **0.011** | <0.01 | 0.157 | <0.01 | 0.508 |  |
|  |  | **Genus-level** |  |  |  |  |  |  |  |  |  |  |  |  |  |  |  |  |  |  |  |  |  |
|  |  | Secretion microbiota | 0.02 | 0.367 | (-0.08)-0.11 | 0.02 | 0.382 | (-0.09)-0.10 | -0.02 | 0.559 | (-0.05)-0.02 | **0.19** | **0.043** | **0.01-0.27** | 0.01 | 0.927 | <0.01 | 0.957 | -0.01 | 0.858 | 0.19 | 0.067 |  |
|  |  | Phylogeny |  |  |  |  |  |  |  |  |  |  |  |  | <0.01 | 0.447 | <0.01 | 0.432 | <0.01 | 0.911 | <0.01 | 0.353 |  |
|  | Cavity | **ASV-level** |  |  |  |  |  |  |  |  |  |  |  |  |  |  |  |  |  |  |  |  |  |
|  |  | Secretion microbiota | -0.09 | 0.785 | (-026)-0.01 | -0.07 | 0.718 | (-0.25)-0.04 | **0.30** | **0.024** | **0.12-0.43** | **0.37** | **0.029** | **0.14-0.53** | -0.14 | 0.393 | -0.10 | 0.511 | **0.30** | **0.024** | **0.30** | **0.028** |  |
|  |  | Phylogeny |  |  |  |  |  |  |  |  |  |  |  |  | **<0.01** | **<0.001** | **<0.01** | **<0.001** | <0.01 | 0.205 | **<0.01** | **0.794** |  |
|  |  | **Genus-level** |  |  |  |  |  |  |  |  |  |  |  |  |  |  |  |  |  |  |  |  |  |
|  |  | Secretion microbiota | 0.10 | 0.227 | (-0.01)-0.24  0 | 0.08 | 0.258 | (0.02)-0.21 | **0.42** | **0.003** | **0.28-0.53** | **0.42** | **0.018** | **0.19-0.58** | 0.04 | 0.729 | 0.04 | 0.790 | **0.51** | **0.001** | **0.31** | **0.022** |  |
|  |  | Phylogeny |  |  |  |  |  |  |  |  |  |  |  |  | <0.01 | <0.001 | <0.01 | <0.001 | <0.01 | 0.394 | <0.01 | 0.474 |  |
|  | Non- cavity | **ASV-level** |  |  |  |  |  |  |  |  |  |  |  |  |  |  |  |  |  |  |  |  |  |
|  |  | Secretion microbiota | 0.19 | 0.072 | 0.09-0.34 | 0.20 | 0.075 | 0.08-0.31 | -0.02 | 0.601 | (-0.10)-0.10 | 0.18 | 0.116 | (-0.10)-0.30 | 0.29 | 0.175 | 0.35 | 0.130 | -0.02 | 0.827 | 0.17 | 0.207 |  |
|  |  | Phylogeny |  |  |  |  |  |  |  |  |  |  |  |  | **<0.01** | **0.009** | **<0.01** | **0.011** | <0.01 | 0.676 | **<0.01** | **0.014** |  |
|  |  | **Genus-level** |  |  |  |  |  |  |  |  |  |  |  |  |  |  |  |  |  |  |  |  |  |
|  |  | Secretion microbiota | 0.08 | 0.250 | (-0.12)-0.19 | 0.07 | 0.288 | (-0.12)-0.18 | -0.08 | 0.779 | (-0.15)-0.01 | -0.01 | 0.497 | (-0.18)-0.09 | 0.09 | 0.579 | 0.09 | 0.618 | -0.08 | 0.429 | -0.01 | 0.933 |  |
|  |  | Phylogeny |  |  |  |  |  |  |  |  |  |  |  |  | **<0.01** | **0.004** | **<0.01** | **0.003** | <0.01 | 0.694 | **<0.01** | **0.020** |  |
| **Nestlings** | All samples | **ASV-level** |  |  |  |  |  |  |  |  |  |  |  |  |  |  |  |  |  |  |  |  |  |
|  |  | Secretion microbiota | **0.33** | **0.001** | **0.24-0.41** | **0.35** | **0.001** | **0.25-0.47** | 0.04 | 0.308 | (-0.01)-0.10 | **0.23** | **0.003** | **0.10-0.32** | **0.70** | **<0.001** | **0.82** | **<0.001** | 0.06 | 0.399 | **0.24** | **0.004** |  |
|  |  | Phylogeny |  |  |  |  |  |  |  |  |  |  |  |  | **<0.01** | **<0.001** | **<0.01** | **<0.001** | **<0.01** | **0.002** | **<0.01** | **<0.001** |  |
|  |  | **Genus-level** |  |  |  |  |  |  |  |  |  |  |  |  |  |  |  |  |  |  |  |  |  |
|  |  | Secretion microbiota | **0.35** | **0.001** | **0.24-0.42** | **0.30** | **0.001** | **0.22-0.39** | 0.05 | 0.239 | (-0.01-0.11) | 0.11 | 0.106 | 0.02-0.18 | **0.30** | **<0.001** | **0.24** | **<0.001** | 0.064 | 0.436 | 0.10 | 0.164 |  |
|  |  | Phylogeny |  |  |  |  |  |  |  |  |  |  |  |  | <0.01 | 0.129 | **<0.01** | **0.043** | <0.01 | 0.082 | <0.01 | 0.668 |  |
|  | Cavity | **ASV-level** |  |  |  |  |  |  |  |  |  |  |  |  |  |  |  |  |  |  |  |  |  |
|  |  | Secretion microbiota | **0.45** | **0.001** | **0.33-0.57** | **0.43** | **0.001** | **0.34-0.59** | 0.02 | 0.394 | (-0.03)-0.09 | **0.19** | **0.029** | **0.02-0.30** | **0.83** | **<0.001** | **0.95** | **<0.001** | -0.02 | 0.766 | 0.17 | 0.058 |  |
|  |  | Phylogeny |  |  |  |  |  |  |  |  |  |  |  |  | **<0.01** | **<0.001** | **<0.01** | **<0.001** | **<0.01** | **<0.001** | **<0.01** | **<0.001** |  |
|  |  | **Genus-level** |  |  |  |  |  |  |  |  |  |  |  |  |  |  |  |  |  |  |  |  |  |
|  |  | Secretion microbiota | **0.35** | **0.001** | **0.24-0.44** | **0.29** | **0.001** | **0.20-0.38** | 0.07 | 0.193 | 0.02-0.13 | 0.03 | 0.344 | (-0.06)-0.12 | **0.39** | **<0.001** | **0.40** | **<0.001** | 0.03 | 0.684 | 0.04 | 0.756 |  |
|  |  | Phylogeny |  |  |  |  |  |  |  |  |  |  |  |  | **<0.01** | **<0.001** | **<0.01** | **<0.001** | **<0.01** | **<0.001** | **<0.01** | **<0.001** |  |
|  | Non- cavity | **ASV-level** |  |  |  |  |  |  |  |  |  |  |  |  |  |  |  |  |  |  |  |  |  |
|  |  | Secretion microbiota | -0.17 | 0.886 | (-0.29)-0.02 | -0.18 | 0.899 | (-0.30-0.04) | 0.04 | 0.376 | (-0.12)-0.30 | -0.08 | 0.608 | (-0.2)-0.10 | -0.31 | 0.237 | -0.39 | 0.214 | 0.04 | 0.802 | -0.05 | 0.694 |  |
|  |  | Phylogeny |  |  |  |  |  |  |  |  |  |  |  |  | **<0.01** | **0.001** | **<0.01** | **0.001** | **<0.01** | **0.003** | <0.01 | 0.501 |  |
|  |  | **Genus-level** |  |  |  |  |  |  |  |  |  |  |  |  |  |  |  |  |  |  |  |  |  |
|  |  | Secretion microbiota | -0.12 | 0.715 | (-0.37)-0.03 | -0.14 | 0.778 | (-0.35)-(-0.03) | -0.001 | 0.423 | (-0.16)-0.18 | -0.16 | 0.752 | (-0.29)-(-0.001) | -0.11 | 0.545 | -0.16 | 0.444 | <0.001 | 0.994 | -0.10 | 0.485 |  |
|  |  | Phylogeny |  |  |  |  |  |  |  |  |  |  |  |  | **<0.01** | **0.002** | **<0.01** | **0.001** | **<0.001** | **0.017** | <0.01 | 0.337 |  |

**Table S12.** Results of General Linear Models using source-tracking (FEAST) output. The proportion of the gland-skin bacterial community originated from the gland secretion was used as dependent variable. We explored the effects of age class, species identity (ID) and nest type in two different models. The first model included the species ID and age class as fixed factors, while the interaction between them was analysed in separate models including also the main effects. The effect of nest type was explored separately for adults and nestlings, including the nest type, sampling locality and the species ID nested within nest type as independent variables. Values in bold are those with associated alpha value lower than 0.05. For random factors, LRT is reported instead of the F statistic.

|  | |  |  |  | F/LRT | *df* | p |
| --- | --- | --- | --- | --- | --- | --- | --- |
|  | **Effect of age class** | | | |  |  |  |
|  | |  | Species | | **4.27** | **3, 34.8** | **0.011** |
|  | |  | Age | | 2.84 | 1, 34.6 | 0.101 |
|  | |  | Locality | | 0.15 | 1, 35.2 | 0.704 |
|  | |  | Species*Age | | **5.53** | **3, 30.6** | **0.004** |
|  | |  | Nest (Species*Age) | | 3.72 | 1 | 0.054 |
|  | **Effect of nest type** | | | |  |  |  |
| Adults | |  | Nest type | | 1.29 | 1, 33 | 0.264 |
|  |  |  | Species (nest type) | | 1.84 | 8, 33 | 0.104 |
|  |  |  | Locality | | 1.43 | 1,33 | 0.240 |
| Nestlings | |  | Nest type | | **7.48** | **1, 20.2** | **0.013** |
|  |  |  | Species (nest type) | | **3.40** | **6, 17.6** | **0.021** |
|  |  |  | Locality | | 0.26 | 1, 22.5 | 0.613 |
|  |  |  | Nest(Species(Nest type)) | | 1. 38 | 1 | 0.240 |
